# Integrated Proteomic and Metabolomic Analyses Show Differential Effects of Glucose Availability in Marine *Synechococcus* and *Prochlorococcus*

**DOI:** 10.1128/spectrum.03275-22

**Published:** 2023-02-01

**Authors:** José Ángel Moreno-Cabezuelo, Guadalupe Gómez-Baena, Jesús Díez, José Manuel García-Fernández

**Affiliations:** a Departamento de Bioquímica y Biología Molecular-Campus de Excelencia Agroalimentaria CEIA3, Universidad de Córdoba, Cordoba, Spain; University of Mississippi

**Keywords:** proteomics, metabolomics, *Prochlorococcus*, marine *Synechococcus*, glucose, mixotrophy

## Abstract

We compared changes induced by the addition of 100 nM and 5 mM glucose on the proteome and metabolome complements in *Synechococcus* sp. strains WH8102, WH7803, and BL107 and *Prochlorococcus* sp. strains MED4, SS120, and MIT9313, grown either under standard light conditions or in darkness. Our results suggested that glucose is metabolized by these cyanobacteria, using primarily the oxidative pentoses and Calvin pathways, while no proof was found for the involvement of the Entner-Doudoroff pathway in this process. We observed differences in the effects of glucose availability, both between genera and between *Prochlorococcus* MED4 and SS120 strains, which might be related to their specific adaptations to the environment. We found evidence for fermentation in *Prochlorococcus* sp. strain SS120 and *Synechococcus* sp. strain WH8102 after 5 mM glucose addition. Our results additionally suggested that marine cyanobacteria can detect nanomolar glucose concentrations in the environment and that glucose might be used to sustain metabolism under darkness. Furthermore, the KaiB and KaiC proteins were also affected in *Synechococcus* sp. WH8102, pointing to a direct link between glucose assimilation and circadian rhythms in marine cyanobacteria. In conclusion, our study provides a wide overview on the metabolic effects induced by glucose availability in representative strains of the diverse marine picocyanobacteria, providing further evidence for the importance of mixotrophy in marine picocyanobacteria.

**IMPORTANCE** Glucose uptake by marine picocyanobacteria has been previously described and strongly suggests they are mixotrophic organisms (capable of using energy from the sun to make organic matter, but also to directly use organic matter from the environment when available). However, a detailed analysis of the effects of glucose addition on the proteome and metabolome of these microorganisms had not been carried out. Here, we analyzed three *Prochlorococcus* sp. and three *Synechococcus* sp. strains which were representative of several marine picocyanobacterial clades. We observed differential features in the effects of glucose availability, depending on both the genus and strain; our study illuminated the strategies utilized by these organisms to metabolize glucose and showed unexpected links to other pathways, such as circadian regulation. Furthermore, we found glucose addition had profound effects in the microbiome, favoring the growth of coexisting heterotrophic bacteria.

## INTRODUCTION

*Prochlorococcus* and marine *Synechococcus* are the two most abundant primary producers on our planet. According to recent estimations, their global populations are ~1.6 × 10^27^ and 6.8 × 10^26^, respectively (1). It is currently believed that global warming will induce the growth of these populations (2, 3), allowing strains adapted to warm waters to expand their habitats to occupy areas closer to the poles as temperatures increase. For decades, these microorganisms were considered phototrophic, especially in the case of *Prochlorococcus*, due to its small genome size, leading to its description as a minimal genome for a phototroph (4). However, recent research has shown multiple lines of evidence indicating that marine picocyanobacteria can take up organic compounds, such as amino acids (5), phosphonates (6), ATP (7), dimethylsulfoniopropionate (8), or glucose (9). Therefore, these organisms seem to behave actually as mixotrophs, being able to take up organic compounds when available in their environment (10). Furthermore, recent studies have shown that mixotrophy is essential for the survival of deep *Prochlorococcus* populations in the oceans (11).

Our team demonstrated that several *Prochlorococcus* strains can take up glucose in laboratory cultures and that glucose addition induced changes in the expression of some genes related to glucose utilization, including *glcH* (9). The gene *glcH* encodes a very-high-affinity, biphasic glucose transporter (12) which can be used to scavenge nanomolar glucose concentrations in the ocean (12) following diel oscillations, with a different timing with respect to the total microbial population (13). We observed a remarkable diversity in the glucose uptake kinetics and *glcH* phylogeny in marine picocyanobacteria (14), suggesting these microorganisms have been subjected to evolutive selection. Furthermore, *glcH* expression is affected by glucose concentration and light availability (15), indicating that the GlcH transporter is used according to the needs of these microorganisms.

In an earlier study, we characterized the effect of the addition of nanomolar concentrations of glucose on the proteome of *Prochlorococcus* SS120 cells (14), and we observed minor changes which suggested that *Prochlorococcus* remained functioning as an autotrophic organism under those conditions. No study has addressed the effect on the proteome of the availability of higher concentrations of glucose. Furthermore, the effect of glucose availability on the metabolome has never been studied, at any glucose concentration. Hence, the glucose metabolization pathway in marine cyanobacteria remains unknown, despite some interesting *in silico* analyses previously published (9, 16). In the current study, we explored these important issues by supplying six representative strains of marine picocyanobacteria (*Prochlorococcus* sp. strains MED4, SS120, and MIT9313 and *Synechococcus* sp. strains WH7803, WH8102, and BL107) with concentrations of glucose in the micromolar and millimolar range, grown under standard illumination conditions or under darkness. Samples of these cultures were then analyzed by quantitative proteomics and metabolomics to provide an integrated view of glucose metabolization in *Prochlorococcus* and marine *Synechococcus*.

Given that natural *Prochlorococcus* and *Synechococcus* populations coexist with heterotrophic bacteria in the oceans, we utilized nonaxenic cultures as a proxy to assess the effect of glucose addition on the microbial community. Proteomic results were analyzed by assigning the identified peptides to the corresponding genus of either cyanobacteria or heterotrophic bacteria, and quantitative results were used to compare community dynamics in the presence of glucose. These results were then compared to the metabolomic changes observed under the same conditions for each picocyanobacterial strain. Our results revealed the differential effects of glucose addition on *Prochlorococcus* versus *Synechococcus* and provided interesting information to understand the metabolic strategies to assimilate glucose utilized by both genera.

## RESULTS

The aim of the present study was to compare the effect of glucose availability on the proteome and metabolome of several model marine cyanobacterial strains from the genera *Synechococcus* and *Prochlorococcus*. We used the same experimental workflow in all cases, by performing triplicate experiments of glucose addition (100 nM and 5 mM) to cultures of *Prochlorococcus* strains MED4, SS120, and MIT9313 and *Synechococcus* strains WH7803, WH8102, and BL107. The effect of two very different glucose concentrations was evaluated: 100 nM, which is close to the glucose concentration observed in the oceans (12), and 5 mM which, although very distant from natural glucose concentrations, was chosen to study how excessive glucose availability affects the metabolism of marine picocyanobacteria (as has been studied in other marine strains [17]). In addition, we assayed the effect of darkness on the glucose utilization pathways.

Selected strains were representative of different clades and ecological adaptations: MED4 belongs to the HLI clade, is adapted to grow near the surface, under high-light conditions (18–20); SS120 belongs to the LLII/III clade and is one of the most recently evolved *Prochlorococcus* strains and is adapted to grow at depth, under low-light conditions (4, 21); MIT9313 belongs to LLIV clade and is also adapted to grow at depth, being one of the oldest strains in the *Prochlorococcus* genus (19, 20). For *Synechococcus*, we selected strain WH8102 (subcluster 5.1, clade IIIa), which is adapted to live in warm, oligotrophic oceans (22), WH7803 (subcluster 5.1, clade V), which shows a general distribution and is one of the most studied marine *Synechococcus* strains (23), and finally the strain BL107 (subcluster 5.1, clade IVa), which was isolated near the coast and is adapted to copiotrophic conditions (24).

### Proteomic analysis.

**FIG 1:**
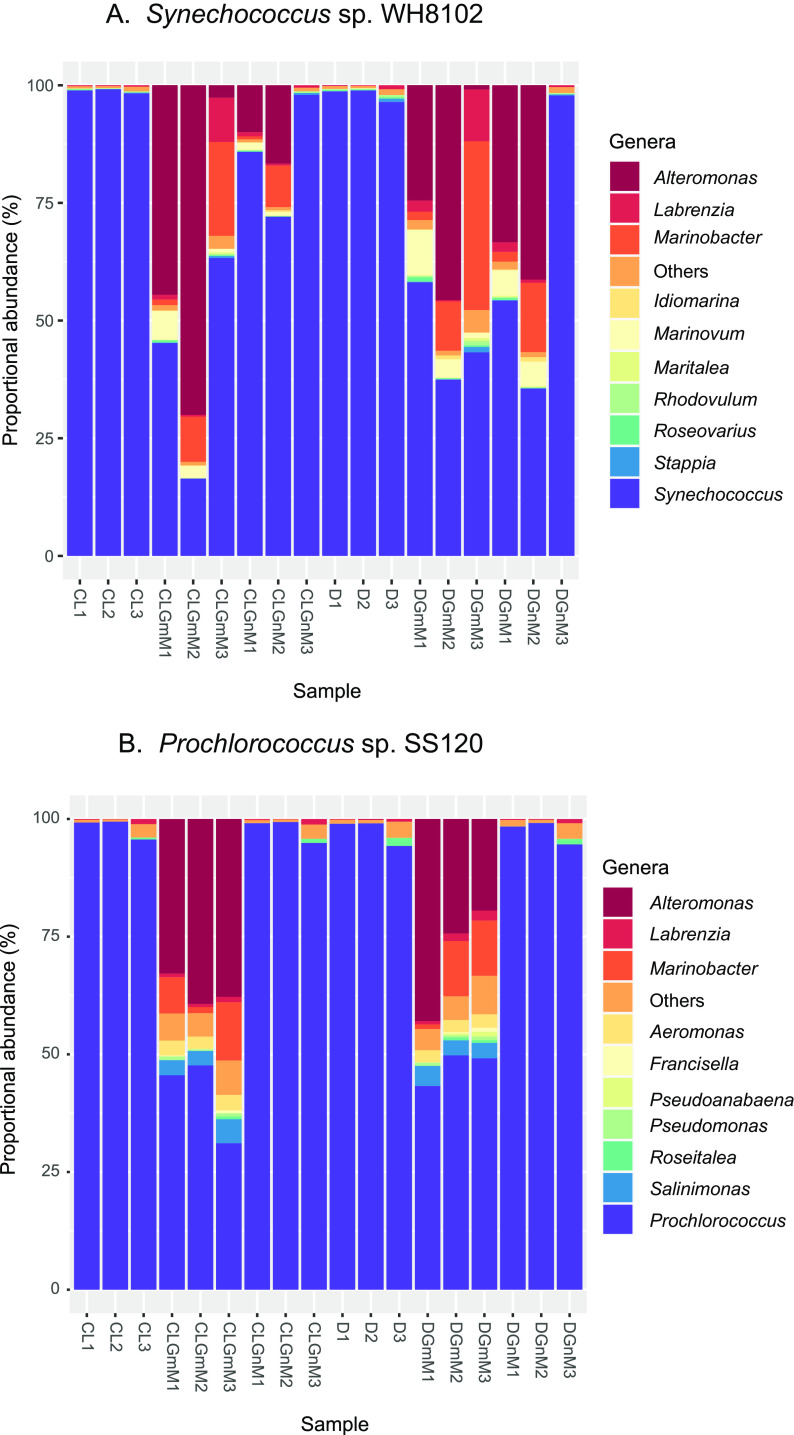
Effect of glucose availability on the proportional abundance of genera in cultures from *Synechococcus* and *Prochlorococcus*. (A) *Synechococcus* sp. WH8102 cultures. (B) *Prochlorococcus* sp. SS120 cultures. Proportional abundance was calculated based on total protein intensity per genus, obtained by relative label-free abundance using the MarRef v6 as database. Each column represents individual samples. CL, control light; D, darkness; GmM, glucose at 5 mM; GnM, glucose at 100 nM. Three biological replicates were prepared per condition.

**(i) Effect of glucose addition on the microbial community in cyanobacterial cultures.** The addition of a sugar molecule, such as glucose, which is readily assimilable by heterotrophic bacteria, was expected to induce significant changes in the relative abundance of the microbial communities in the *Prochlorococcus* and *Synechococcus* cultures, which are nonaxenic. Under phototrophic conditions, these cultures tend to have low levels of heterotrophic bacteria, but that situation changes significantly upon glucose addition. For this reason, as a strategy to quantify the impact of glucose amendment, we calculated the proportional abundance of each genus by searching our proteomics data against an extensive database and normalizing the abundance to the total protein intensity in the sample, as described in Materials and Methods (Fig. 1; see also Fig. S1 in the supplemental material).

Proportional abundance showed that very low levels of heterotrophic bacteria (0.86 to 3.56%) were quantified in *Synechococcus* sp. strain WH8102 control cultures (with no glucose addition), grown either under standard illumination conditions or under darkness (Fig. 1A). Glucose addition induced a clear increase in the abundance of some genera, and this was particularly remarkable for *Alteromonas*, *Marinobacter*, and *Marinovum*. This increase was greater in cultures with millimolar concentrations of glucose compared to those with nanomolar glucose addition, although we observed significant variability among replicates. In cultures growing under darkness, the increase in heterotrophic populations was similar to the increase observed in light; however, heterotrophic genera reached higher levels after nanomolar glucose addition in two of the cultures subjected to darkness compared to those in the light, suggesting that *Synechococcus* WH8102 populations grown in the light were more competitive due to the energy provided by light.

In the case of *Synechococcus* sp. strain WH7803 (Fig. S1A), the effect of glucose amendment on heterotrophic abundance was similar to that for *Synechococcus* WH8102, but there was no appreciable difference between cultures grown in light or dark conditions: for each glucose concentration, the increased heterotrophic abundance was comparable, regardless of the light condition. This could point to a lower competitiveness in *Synechococccus* sp. WH7803 with respect to WH8102 under phototrophic conditions. In these experiments, the main heterotrophic groups were *Alteromonas*, *Labrenzia*, and *Marinobacter*.

*Synechococcus* sp. strain BL107 (Fig. S1B) showed higher levels of heterotrophic bacteria in cultures even with no glucose addition, compared to the other two studied *Synechococcus* strains. Under these circumstances, addition of either glucose concentration, either in the light or under darkness, promoted a remarkable increase of heterotrophic populations, especially of *Alteromonas*, which became the dominant heterotrophic bacterium in several of the samples. The genera *Roseovarius* and *Thalassospira* were particularly abundant in BL107 cultures, unlike in the two other studied *Synechococcus* strains.

The results with *Prochlorococcus* SS120 (Fig. 1B) were similar to those observed for *Synechococcus* WH8102, with low levels of contamination in control cultures (0.8 to 5%). Interestingly, addition of nanomolar glucose induced almost no increase in the levels of heterotrophic bacteria, as it was detectable only in cultures with millimolar glucose, unlike the case of *Synechococcus* WH8102. The main genera of heterotrophic bacteria were the same observed in *Synechococcus* cultures (*Alteromonas* and *Marinobacter*) and *Salinimonas*.

Finally, the results with *Prochlorococcus* MED4 (Fig. S1C) were similar to those with *Synechococcus* WH7803, with higher levels of basal contamination that were significantly increased upon glucose addition, and this was especially remarkable at the higher sugar concentration.

**(ii) Effects of glucose addition on the proteomes of *Prochlorococcus* and *Synechococcus* under light versus dark conditions.** A label-free quantitative proteomics approach allowed the quantification of a meaningful fraction of the protein complement in the studied strains. After compiling results from all the samples analyzed, a total of 2,684 protein groups were quantified for the main pathways in cyanobacteria metabolism (Table S1). Figure S2 shows global pathway overviews of quantified proteins in *Synechococcus* sp. strains WH8102 and WH7803 and *Prochlorococcus* sp. strains SS120 and MED4. Detailed quantitative results are available in Table 1 and Tables S1 and S2. All of the protein differences described in the following paragraphs met the statistically significant difference criteria outlined below in Materials and Methods.

**TABLE 1:** Changes induced by glucose addition to *Synechococcus* and *Prochlorococcus* cultures growing under light or dark conditions^*a*^

Protein (gene)	Pathway	WH8102+mM Gluc L/D+nM Gluc L/D	WH7803+mM Gluc L/D+nM Gluc L/D	BL107+mM Gluc L/D+nM Gluc L/D	MED4+mM Gluc L/D+nM Gluc L/D	SS120+mM Gluc L/D+nM Gluc L/D
Glucokinase (*glk*)	EMP	ns/2.73 0.8/ns	ns	-	-	ns/5.35 ns/ns
Glucose-6-phosphate isomerase (*pgi*)	EMP	1.68/1.39 ns/ns	ns	ns	-	ns
Phosphofructokinase (missing gene)	EMP	-	-	-	-	-
Fructose-1,6-bisphosphate aldolase (*fba*)	EMP	2.05/ns ns/ns	ns	ns	ns	0.37/0.39 ns/ns
Triose phosphate isomerase (*tpiA*)	EMP	ns	ns/2.32 0.59/ns	-	ns	ns
Glucose 1 dehydrogenase (*gdh*)	EDD/OPP	-	ns		-	-
6-Phospho-gluconolactonase (*pgl*)	EDD/OPP	ns	ns	-	-	ns/2.64 ns/ns
Glucose-6-P dehydrogenase (*zwf*)	EDD/OPP	ns/4.92 ns/ns	-	-	-	ns
Glucose-6-P dehydrogenase effector OpcA	EDD/OPP	2.47/ns ns/ns	-		ns	
2-Keto-3-deoxy-phosphogluconate aldolase (*eda*)	EDD	-	-	-	-	ns/0.025 ns/ns
6-Phospho-gluconate dehydrogenase (*gnd*)	OPP	ns	-		ns	ns/1.42 ns/ns
Ribose-5-phosphate isomerase (*rpi*)	OPP and Calvin	ns/1.67 ns/ns	ns/1.51 0.94/ns	ns	ns	ns
Ribulose phosphate 3 epimerase (*rpe*)	OPP and Calvin	ns/ns ns/ns	1.32/ns 1.13/ns	1.6/ns ns/ns	-	ns
Transaldolase (*tal*)	OPP	2.12/2.48 ns/ns	2.19/ns ns/1.36	2.58/2.49 ns/ns	0.69/ns ns/0.94	1.47/2.67 ns/ns
Transaketolase (*cbbT/tkt*)	OPP	1.91/ns ns/ns	ns/ns ns/ns	2.54/ns ns/ns	ns	ns
Glyceraldehyde 3-phosphate dehydrogenase (*gap1*)	COM		ns/0.74 ns/ns	ns		
Glyceraldehyde 3-phosphate dehydrogenase (*gap2*)	COM	ns	-	-	ns	0.48/0.68 ns/ns
Glyceraldehyde 3-phosphate dehydrogenase (*gap3*)	COM	ns	-	-	-	10.17/7.17 ns/ns
Phosphoglycerate kinase (*pgk*)	COM	ns	ns		ns	0.34/0.43 ns/ns
Phosphoglycerate mutase (*gpm*)	COM	ns	-	-		ns/1.99 ns/ns
Enolase (*eno*)	COM	ns/2.15 ns/ns	-	-	ns	ns/ns ns/0.86
Pyruvate kinase (*pyk*)	COM	ns	-	ns	ns	ns
Pyruvate dehydrogenase α subunit (*pdhA*)	COM	2.4/2.11 ns/ns	3.14/2.73 ns/ns	-	ns	ns/1.56 1.12/ns
Pyruvate dehydrogenase β subunit (*pdhB)*	COM	2.83/ns ns/ns	ns		ns	ns
Dihydrolipoamide acetyltransferase of pyruvate dehydrogenase (*pdhC*)	COM	ns/ns 1.82/ns	ns	ns/ns 5.7/ns	ns	ns
Acetyl-CoA synthetase (*acs*)	Alt synt Pyr	2.62/ns ns/ns	ns	-	ns	ns
Citrate synthase (*gltA*)	Krebs	1.49/ns ns/ns	-	-	ns	2.62/ns ns/ns
Fructose-1,6-bisphosphatase (*fbp*)	Gluconeo/Calvin	1.82/1.42 ns/ns	ns	ns	ns	ns/0.68 ns/ns
Glycerol kinase	FAD	1.98/1.75 ns/ns	-	2.23/4.94 ns/ns	-	-
α-1,4-Glucan phosphorylase (*glgP*)	Glycogen	4.82/ns ns/ns	-	-	ns	2.37/ns ns/1.12
Glycogen debranching enzyme (*pulA*)	Glycogen	-	-	-	-	ns
Glycogen synthase (*glgA*)	Glycogen	-	-	-	ns	ns/2.70 ns/ns
1,4-α-Glucan branching enzyme (*glgB*)	Glycogen	ns	1.47/1.66 ns/ns	ns	ns	ns/2.03 ns/ns
Isocitrate dehydrogenase (*icd*)		ns	-	ns	ns	-
KaiA	Clock	-	ns	-	-	-
KaiB	Clock	ns/1.87 ns/1.56	-	-	-	ns
KaiC	Clock	3.08/ns 1.59/2.75	ns	-	ns	ns
Circadian phase modifier CpmA	Clock	ns/1.68 ns/ns	-	-	-	ns
Phosphoenolpyruvate carboxylase (*ppc*)	Gluconeo	ns/27.55 ns/ns	-	-	ns	1.48/1.70 ns/ns
Phosphoglucomutase (*pgm*)	Glycogen deg	ns/1.29 1.18/ns	ns	ns/3.04 ns/ns	ns/1.47 ns/ns	ns
Phosphoribulokinase (*prk*)	Calvin	ns/1.57 ns/ns	ns/1.07 1.06/ns	1.95/1.80 ns/ns	ns/ns ns/0.88	ns/1.9 ns/ns
PII		1.45/ns 1.55/ns	0.85/ns ns/ns	ns	ns	0.78/ns ns/ns
RuBisCO large subunit	Calvin	ns/1.19 ns/ns	ns	ns/ns 0.48/ns	ns	ns
RuBisCO small subunit	Calvin	2.08/ns ns/ns	1.52/1.69 ns/0.92	2.79/4.05 ns/ns	-	ns
Sugar fermentation-stimulating protein		ns/2.09 ns/ns	-	-	-	ns/1.8 ns/ns

a The table shows significant fold change values for the indicated proteins under the indicated condition. Within each cell, the top row numbers correspond to the relative change in protein concentration in cultures subjected to 5 mM glucose addition in light or darkness, compared to a control grown with no added glucose and the same light conditions; the bottom numbers show the same comparison in cultures subjected to 100 nM glucose. ns, non-significant changes. -, the protein was non identified. Proteins are classified by pathways: EMP, Embden-Meyerhof-Parnas pathway; EDP, Entner-Dodouroff pathway; OPP, oxidative pentose phosphate pathway; COM, enzymes common to the three glycolytic pathways.

*(a) Enrichment analysis.* In *Prochlorococcus* SS120, 224 proteins increased in abundance when 5 mM glucose was provided to cultures growing in the dark. Using DAVID functional annotation clustering, we found that these proteins were grouped in 5 significant clusters. These significant clusters entailed terms related to cytoplasm (enrichment score, 8.84), protein biosynthesis (enrichment score, 3.55), ATP binding (enrichment score, 2.37), amino acid synthesis (enrichment score, 2.17), and pyridoxal phosphate-related transferase (enrichment score, 1.32). By contrast, proteins whose abundance decreased (65 proteins) were grouped in two significant clusters: cluster 1 was related to the term ribosome proteins (enrichment score, 1.34), and cluster 2 was related to ATP synthesis (enrichment score, 1.03). These results seemed to indicate that *Prochlorococcus* cultures continued growing when glucose was provided in the dark.

Do *Prochlorococcus* cells respond in the same way when glucose is added to cultures growing with light availability? A total of 163 proteins, whose abundance increased after adding 5 mM glucose to cultures kept in the light, were grouped in two clusters: cluster 1 was related to pyridoxal phosphate and cluster 2 was related to tropism domain, a domain related to DNA binding. Interestingly, proteins whose abundance decreased were grouped in the same two clusters as were obtained in the dark: cluster 1 was related to the term ribosome proteins (enrichment score, 3.11), and cluster 2 was related to ATP synthesis (enrichment score, 0.89).

In *Synechococcus* WH7803, proteins with decreased abundance after 5 mM glucose addition (either in the light or dark) did not result in any significant clusters. By contrast, proteins whose abundance increased could be grouped in 2 significant clusters. Both in the dark and in the light, the most significant cluster included terms related to antenna complex (scores of 1.61 in darkness and 1.75 in the light). The second cluster comprised terms related to cytoplasm (scores of 0.73) in darkness, while in light the second cluster enriched terms included ribosomes (score of 1.37). This suggested that glucose addition stimulated the production of photosynthetic proteins which were harvesting light.

In *Prochlorococcus* MED4, *Synechococcus* WH8102, and *Synechococcus* BL107, regrettably, UniProt accession numbers have not been well mapped in DAVID, and our searches delivered nonsignificant or “not confident” results.

**FIG 2:**
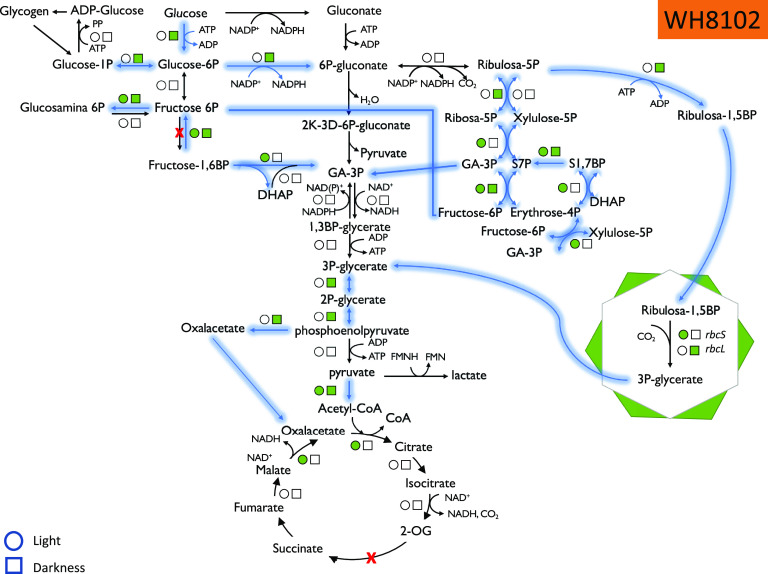
Summary of the proteomics results, showing the statistically significant changes in enzymes involved in glucose metabolism in *Synechococcus* sp. strain WH8102 under light and dark conditions. Circles, light; squares, darkness. Green color indicates an increase in abundance. Arrows indicate the metabolic flow upon glucose addition.

**FIG 3:**
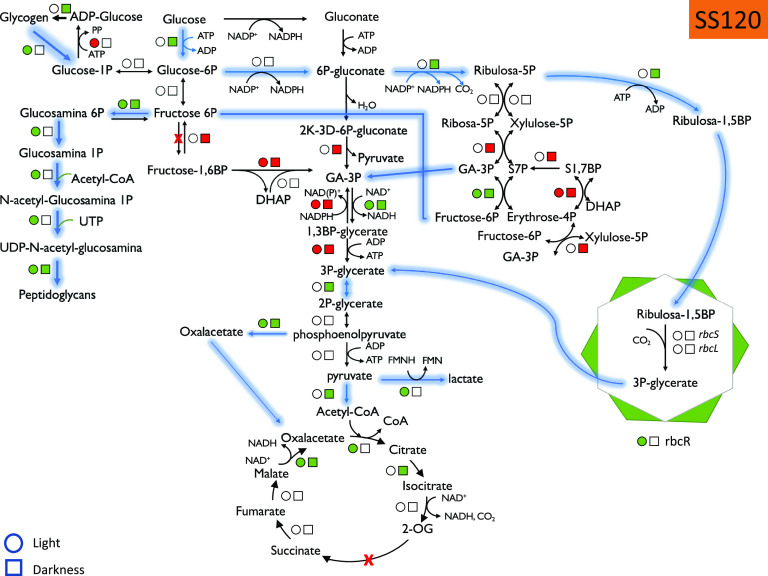
Summary of the proteomics results, showing the statistically significant changes in enzymes involved in glucose metabolism in *Prochlorococcus* sp. strain SS120 under light and dark conditions. Circles, light; squares, darkness. Green color indicates an increase in abundance, and red color indicates a decrease in abundance. Arrows indicate the metabolic flow upon glucose addition.

*(b) Glucose assimilation pathways.* The quantitative data allowed us to explore the effect of glucose addition on the abundance of enzymes belonging to the main pathways related to glucose metabolism, among others (Fig. 2 and 3; Fig. S3). A common feature to all strains analyzed was that, although changes were not dramatic, a higher number of proteins showed significant changes in abundance after the addition of 5 mM glucose compared to those after 100 nM glucose addition.

Genomic information showed that *Prochlorococcus* and marine *Synechococcus* strains possess genes encoding the enzymes required to assimilate glucose by using the pentoses phosphate pathway, but not by the glycolysis pathway (since both genera lack the genes encoding phosphofructokinase) (9, 16). More recently, the Entner-Doudoroff pathway has been proposed to be also involved in glucose assimilation in *Prochlorococcus* (16). Hence, we analyzed the changes in proteins involved in these pathways.

The responses were not uniform in the five studied picocyanobacteria strains; however, it was clear that many of the enzymes showed increases upon glucose addition, particularly at the higher glucose concentration (Fig. 2 and 3; Fig. S3). For instance, all 19 enzymes whose abundance changed in *Synechococcus* sp. strain WH8102 (Fig. 2) were increased in *Prochlorococcus* sp. strain SS120, with 20 enzymes increased in abundance while only 10 decreased in abundance (Fig. 3).

An interesting aspect of our results is the lack of evidence supporting the involvement of the Entner-Doudoroff pathway in the glucose metabolism by marine picocyanobacteria: we could only detect 2-keto-3-deoxyphosphogluconate aldolase (an enzyme specific to this pathway) in *Prochlorococcus* strain SS120 (Q7VDW4), and surprisingly, it decreased 0.025-fold upon glucose addition under darkness with respect to the controls grown in the dark with no glucose addition.

Glucose-6-phosphate dehydrogenase (G6PDH) is a key enzyme in glucose metabolism and is subject to fine regulation; changes observed in our experiments suggested that glucose addition triggered the metabolic machinery required for its utilization. Both the OpcA protein, the G6PDH effector, and G6PDH itself increased in abundance in strain WH8102. OpcA increased 2.47-fold when glucose was added in millimolar concentrations to cultures growing under standard light conditions, and G6PDH showed the maximum fold change (4.92 times) when glucose in millimolar concentrations was added to cultures growing under darkness. In contrast, G6PDH abundance showed no significant changes in *Prochlorococcus* SS120.

When we analyzed the enzymes related to the pentose phosphate pathway, we observed some proteins whose abundance increased after glucose addition: in *Synechococcus*, ribose-5-phosphate isomerase (increases of 1.50-fold in WH7803 and 1.67-fold in WH8102), ribose-5-phosphate epimerase (increases of 1.32-fold in WH7803 and 1.59-fold in WH8102), transaldolase (about 2-fold increase in all three strains), and transketolase (1.91-fold in WH8102 and 2.53-fold in BL107). Those changes suggested the involvement of the pentose phosphate pathway in the metabolism of glucose in *Synechococcus*. Interestingly, we also observed a 2-fold increase in fructose-1,6-bisphosphate aldolase and fructose-1,6-bisphosphatase in *Synechococcus* WH8102. Both enzymes belong to the glycolysis pathway in freshwater cyanobacteria, which possess phosphofructokinase. However, since *Prochlorococcus* and marine *Synechococcus* lack the gene encoding this enzyme, both fructose-1,6-bisphosphate aldolase and fructose-1,6-bisphosphatase likely work in the opposite direction, in a cyclic metabolic flow of the pentose phosphate pathway which allows production of reducing power. The concentration of both enzymes decreased in SS120 upon glucose addition. In fact, G6P isomerase also showed an increase after glucose addition in WH8102 (1.67-fold in the light and 1.40-fold in the dark after 5 mM glucose addition). Overall, it seems that *Synechococcus* WH8102 uses the pentose phosphate pathway for glucose metabolism, while SS120 seems to use the Calvin cycle.

*Prochlorococcus* showed some other differences in glucose metabolism compared to *Synechococcus.* For instance, we observed a strong increase for glucokinase in SS120, with a 5.35-fold increase under darkness, in sharp contrast with *Synechococcus* WH8102, where glucokinase showed no significant changes after glucose addition.

With respect to glycogen metabolism, we observed that the enzyme alpha-1,4-glucan phosphorylase increased in abundance in the presence of 5 mM glucose in both *Prochlorococcus* SS120 (2.37-fold) and *Synechococcus* WH8102 (4.82-fold), indicating that glycogen degradation seems to be activated in the presence of glucose when cultures are grown in light. However, for *Prochlorococcus* SS120, glycogen synthesis was activated when cultures were grown in the presence of glucose in the dark, since both glycogen synthase and the branching enzyme increased in abundance about 2-fold.

Furthermore, when comparing the responses of *Prochlorococcus* MED4 and that of strain SS120, we also observed some interesting differences: in MED4, the enzymes involved in the oxidative pentose phosphate pathway showed small changes upon glucose addition, and generally darkness had little effect. However, in SS120, we found a strong upregulation for 6-phospho-gluconolactonase under darkness (2.64-fold). In turn, transaldolase showed small (but statistically significant) increases in SS120 (1.47-fold in light, 2.67-fold under darkness), while in MED 4 we observed a decrease (0.69-fold in light).

Some ribulose-5-phosphate metabolism seems to deviate to the Calvin cycle in both *Prochlorococcus* and *Synechococcus*, as we observed an increase in the abundance of phosphoribulokinase and the small and large subunits from RubisCO. In addition, the RubisCO operon transcriptional regulator increased in abundance 6 times when glucose was present under light conditions for strain SS120.

In the lower parts of glucose-metabolizing pathways (which are common, regardless of glucose metabolism occurs via glycolysis, the Entner-Doudoroff pathway, or pentose phosphate pathway), we found some relevant changes as well. In the case of *Prochlorococcus*, once again we found a striking contrast in the results for MED4 versus SS120. While MED4 showed only minor changes in most of the enzymes, SS120 showed significant changes for glyceraldehyde-3-phosphate dehydrogenase. In *Prochlorococcus* SS120, glyceraldehyde-3-phosphate dehydrogenase *gap2* decreased in abundance (0.47-fold in light and 0.67-fold in darkness), while glyceraldehyde-3-phosphate dehydrogenase *gap3* showed a strong increase upon glucose addition (10.16-fold in light and 7.16-fold in darkness). Phosphoglycerate mutase, enolase, and the α and β subunits of pyruvate dehydrogenase increased in both *Prochlorococcus* and *Synechococcus* strains.

*(c) Krebs cycle and fermentation pathways.* Some enzymes from the Krebs cycle also showed significant increases, for instance, citrate synthase in both strain SS120 (Q7VE29; 2.61-fold) and strain WH8102 (Q7U401; 1.49-fold) grown under light conditions as well as malate:quinone oxidoreductase, which showed a strong increase in both SS120 (Q7VDF8; 49.1-fold under light and 29.3-fold under darkness conditions) and WH8102 (Q7U5L7; 19.68-fold under light conditions). It is interesting that the enzyme phosphoenolpyruvate carboxylase showed an increase that was particularly strong in strain WH8102 of *Synechococcus* (Q7U4M0; 27.55-fold increase when grown under dark conditions).

Fermentation of glucose seemed to be stimulated in our experiments. First, the abundances of the sugar fermentation stimulation proteins were elevated under darkness in both *Prochlorococcus* SS120 (Q7VDS4; 1.72-fold) and *Synechococcus* WH8102 (Q7U9K2; 2-fold). Second, and most importantly, l-lactate dehydrogenase was about 9 times more abundant when glucose 5 mM was added under light conditions, compared to control cultures grown under the same conditions.

Beyond the changes observed in pathways related to energy metabolism or glucose metabolism, we found some glucose-induced proteomic alterations which might be of potential relevance.

*(d) Photosynthesis.* Photosynthesis was one of the pathways affected by the availability of glucose in all analyzed strains. Abundance of photosystem I proteins seemed not to be affected by glucose addition in any of the strains analyzed (Table S1). However, upon glucose addition, the abundance of some proteins related to photosystem II decreased. Examples of these proteins are the 12-kDa extrinsic protein (Q05YG2) from *Synechococcus* BL107; the lipoprotein Psb27 (Q7VD76) in SS120; the Psb28 protein in BL107 (Q066J5); and the manganese-stabilizing polypeptide PsbO from both *Prochlorococcus* sp. SS120 (Q7VDW1) and *Synechococcus* WH8102 (Q7U9F5). The same behavior was shown for some pigments, such as C-phycoerythrin in BL107 (Q05ZA5 and Q05Z96) and WH7803 (A5GJ03) and R-phycoerythrin subunit beta (Q7VDN2) in strain SS120, although R-phycocyanin (subunits alpha and beta) increased about 1.75-fold in WH7803 upon addition of 5 mM glucose. Conversely, we also observed an increase in the abundance of some other proteins also related to photosynthesis. For example, some phycobilisome proteins increased in abundance in the presence of glucose (phycobilisome linker polypeptide in *Synechococcus* strain WH7803 [A5GJ12], the phycobilisome linker polypeptide in *Synechococcus* BL107 [Q05Z87], and the phycobilisome linker polypeptide CpeC [Q7U4R5] in WH8102 and the phycobilisome rod-core linker polypeptide CpcG in *Synechococcus* WH8102 [Q7U9E4] and WH7803 [A5GNR7]). In addition, allophycocyanin also increased in abundance in all three strains of *Synechococcus*: WH7803 (A5GND7), WH8102 (Q7U6V7), and BL107 (Q064Q2).

*(e) Circadian rhythms.* We found increases in the circadian proteins KaiB and KaiC (roughly 3-fold for both) in *Synechococcus* sp. strain WH8102. However, the concentrations of both proteins did not show significant changes in *Prochlorococcus* SS120. Interestingly, darkness did not seem to strongly affect these increases in any of the cases described above. Since these proteins constitute the core of the circadian clock in cyanobacteria, our results suggest that glucose addition might affect the circadian regulation of metabolism in these microorganisms. Recent results obtained in field studies of *Prochlorococcus* fit nicely with this hypothesis, indicating that glucose addition might induce a delay in circadian rhythms (13). The adaptive value of those changes, and the physiological mechanism underlying them, remain to be studied.

Kai proteins are subjected to circadian phosphorylation cycles (25), and therefore we were interested in checking possible posttranslational phosphorylation in the Kai proteins detected in our proteomic data set. It is important to note that we performed fragmentation using collision-induced dissociation, and therefore it is very likely that putative phosphorylations of peptides were lost, since phosphorylation is quite labile. However, since sometimes it is still possible to see such modifications on the peptides, we performed an analysis of the identification of posttranslational modifications in peptides from Kai proteins by using the Peaks PTM tool. This analysis showed that none of the isoforms identified were phosphorylated under the conditions of our experiments.

*(f) Signal recognition.* In SS120, the signal recognition particle protein Q7VAU4, which is present in most strains of *Prochlorococcus* and *Synechococcus*, showed an increased concentration with the high glucose concentration. The function of this protein might be related to the recognition of glucose at the cell membrane, and it might deserve further investigation in the context of glucose detection by marine cyanobacteria.

Finally, in most strains the phosphate-binding protein PstS, which is also widely distributed among *Prochlorococcus* and *Synechococcus*, showed a strong increase in abundance (almost 15-fold in the SS120) under darkness. PstB, a phosphate import ATP-binding protein, also increased in abundance (9-fold in SS120) as did the phosphate starvation-inducible protein PhoH, suggesting that the transport of phosphate is stimulated in the presence of glucose and is a possible physiological link between phosphate uptake and glucose availability.

**(ii) Effects of glucose availability on the proteome of the main heterotrophic bacteria in cyanobacterial cultures.** The most abundant heterotrophic bacteria in our cultures were the genera *Alteromonas* and *Marinobacter* (Fig. 1; Fig. S1); consequently, we analyzed the changes induced by glucose addition on their proteomes (Tables S3 and S4 describe the proteomic changes in *Alteromonas* and *Marinobacter* grown in the cyanobacterial cultures, respectively). Our results showed a very strong effect, with a series of proteins which suddenly appeared upon glucose addition (especially remarkable in the case of millimolar glucose); additionally, multiple metabolic pathways were affected, especially those related to C metabolism (glycolysis, pentose phosphate pathway, and tricarboxylic acid [TCA] cycle). For instance, phosphoglycerate kinase, transketolase, transaldolase pyruvate kinase, and glucose-6-phosphate isomerase were upregulated in *Alteromonas* from *Synechococcus* WH8102 and BL107, and malate dehydrogenase increased in *Alteromonas* from all tested *Synechococcus* strains. Enzymes linking carbon and nitrogen metabolism (glutamine synthetase and isocitrate dehydrogenase) were also upregulated.

In the case of *Marinobacter* from *Synechococcus* cultures, surprisingly, the enzymes mentioned above did not appear among those which showed the strongest changes. However, we observed increases in isocitrate dehydrogenase and the PII protein, which is involved in the control of C/N metabolism.

For *Alteromonas* growing in *Prochlorococcus* sp. strain SS120 cultures, we also observed clear changes in enzymes related to C metabolism: G6P isomerase, phosphogluconate dehydratase, isocitrate dehydrogenase, phosphoglycerate kinase, phosphoribulokinase, malate dehydrogenase, and pyruvate kinase increased, in many cases with significant values. In *Alteromonas* grown in MED4 cultures, phosphoribulokinase was the most affected protein (more than 100-fold increase in light cultures with millimolar glucose), showing increases as well for G6P dehydrogenase, citrate synthase, phosphoglycerate kinase, and malate dehydrogenase.

In the case of *Marinobacter* from *Prochlorococcus* SS120 cultures, and unlike what we observed in *Synechococcus* cultures, there were clear changes in enzymes related to central routes of C metabolism: phosphoglycerate kinase, glyceraldehyde-3-phosphate dehydrogenase, 6-phosphogluconolactonase, isocitrate dehydrogenase, G6P dehydrogenase, transketolase, citrate synthase, and malate dehydrogenase increased more than 4-fold upon millimolar glucose addition under light conditions. However, in *Marinobacter* from MED4 cultures, we could not detect these enzymes among those which changed most; for instance, malate dehydrogenase increased only 2-fold.

It is worth noting that the results obtained for both *Alteromonas* and *Marinobacter* in light versus darkness (either in control or glucose-amended cultures) were not similar, despite the fact that these organisms are heterotrophic and thus do not depend on light for their metabolism.

### Metabolomic analysis: effects of glucose availability on *Prochlorococcus* and *Synechococcus* strains.

**FIG 4:**
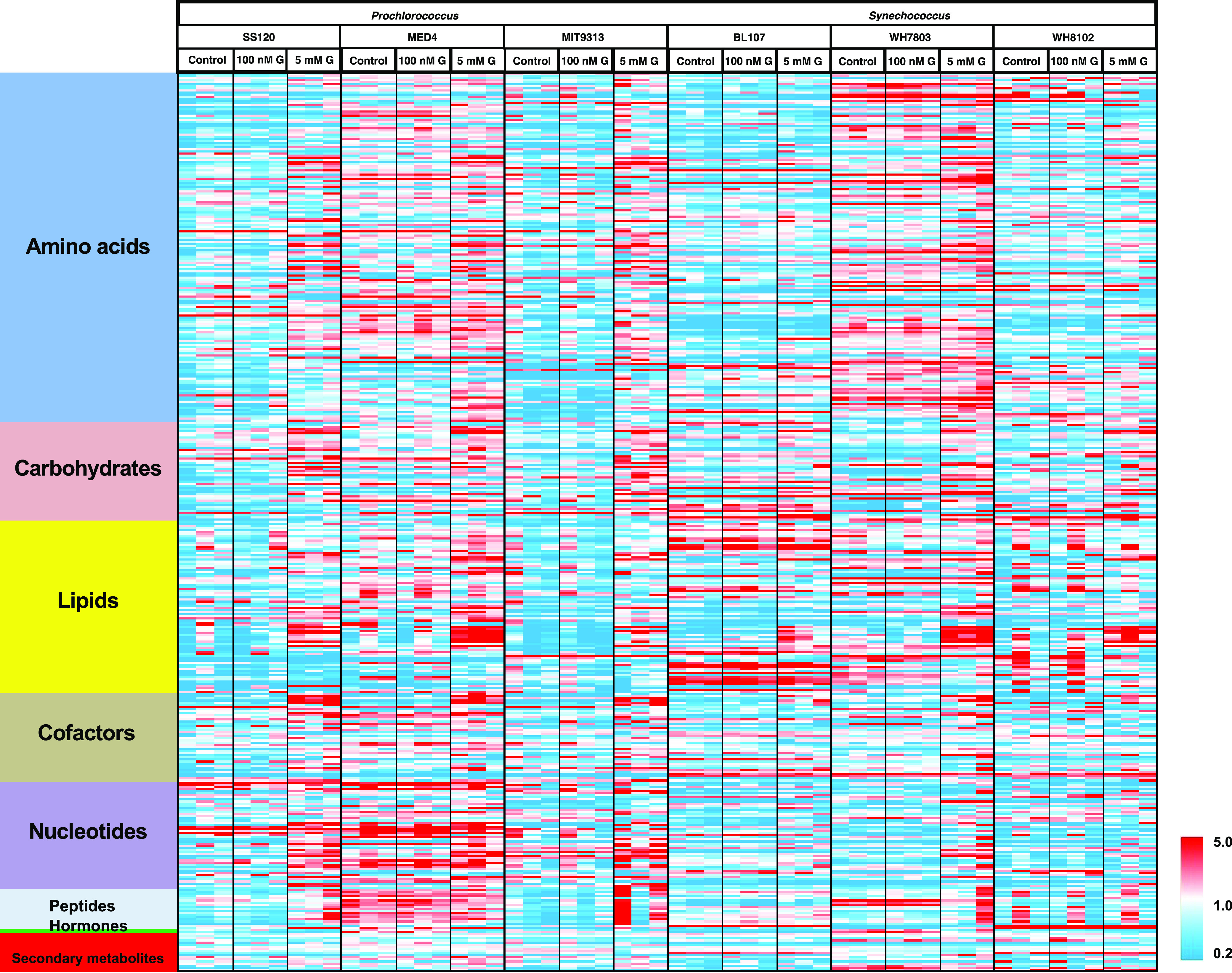
Heatmap showing the main metabolome changes observed in the six studied picocyanobacterial strains. Red color indicates an increase in abundance, and blue color indicates a decrease in abundance, following the scale shown at the bottom right.

All the experiments for metabolomic determinations were carried with cultures in the light. Our results showed that 5 mM glucose addition led to a strong metabolic shift toward overall anabolic patterns in all the studied cyanobacterial cultures. We observed that 469 metabolites increased in concentration with the higher glucose concentration and a decrease of 124 metabolites was produced, while 100 nM glucose had no measurable effect on any of the strains. The patterns of biosynthetic activity induction were stronger in all the *Prochlorococcus* strains, in which most amino acid concentrations were significantly increased in samples subjected to 5 mM glucose, but this pattern was much weaker, and often absent, in *Synechococcus* strains. The BL107 strain was notably the least affected by the provision of the high glucose concentration, while WH7803 showed significantly higher basal pools of metabolites, especially amino acid compounds (Fig. 4).

Amino acid metabolism overall represented a strong differentiator between *Prochlorococcus* and *Synechococcus* cultures. *Prochlorococcus* cultures accumulated much higher levels of most proteinogenic amino acids in the 5 mM glucose samples, but *Synechococcus* strains only showed this effect for a limited number of amino acids (tyrosine, glutamine, glutamate, and cysteine). Asparagine was the exception, in that it was not induced in any of the strains. One of the greatest fold change increases under high glucose was for the strong osmolyte proline, but only in *Prochlorococcus* strains. This was in contrast to the significant induction of trehalose primarily in *Synechococcus* strains, a compound with similar osmolytic properties.

Catabolites of amino acids (as well as nucleotides) were generally lower in the high-glucose samples for several catabolites of tryptophan, tyrosine, arginine, and lysine, such as kynurenine, indole-3-carboxylate, o-tyrosine, 2-oxoarginine, and pipecolate. This reflected the general shift into a more anabolic state.

Several lipids were increased upon glucose addition, especially with the highest glucose concentration, and significantly so in *Prochlorococcus* sp. SS120 and MED4. In *Synechococcus* strains, the most significant changes were found in *Synechococcus* sp. BL107 and WH7803.

Cofactors were increased by the higher glucose concentration, which was again consistent with a more anabolic state. This was more obvious in the *Prochlorococcus* strains and in *Synechococcus* sp. WH7803. Among the cofactors, intermediates in the biosynthetic pathway for coenzyme A (CoA) showed very dramatic induction in the high-glucose samples. CoA is central to a wide range of enzymatic reactions and pathways and serves as the primary acyl carrier in lipid metabolism. Levels of CoA were >60-fold induced in *Prochlorococcus* sp. MED4 and >10-fold induced in all strains except for *Synechococcus* BL107, in which it was only induced 3.5-fold. However, final induced levels in *Prochlorococcus* sp. MIT9313 were lower than in *Synechococcus* sp. BL107.

Biosynthetic precursors for both purines and pyrimidines were strongly induced by high glucose in all cyanobacterial strains. This could suggest that the synthesis of nucleic acids in the cells increased due to the rise in anabolism produced by the addition of glucose. The effects were more pronounced in *Prochlorococcus* strains.

**FIG 5:**
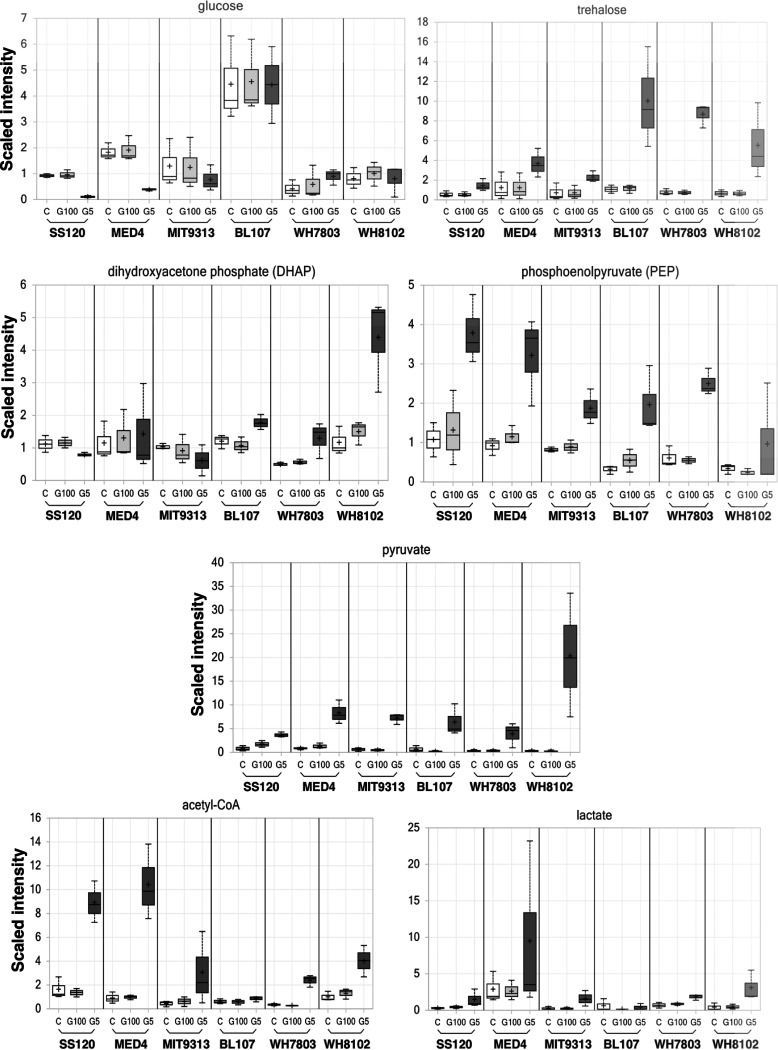
Changes in metabolites related to glycolysis. Scales show a relative quantitation for each metabolite, with bars indicating deviations. White, control culture; light gray, culture with 100 nM glucose; dark gray, culture with 5 mM glucose.

**FIG 6:**
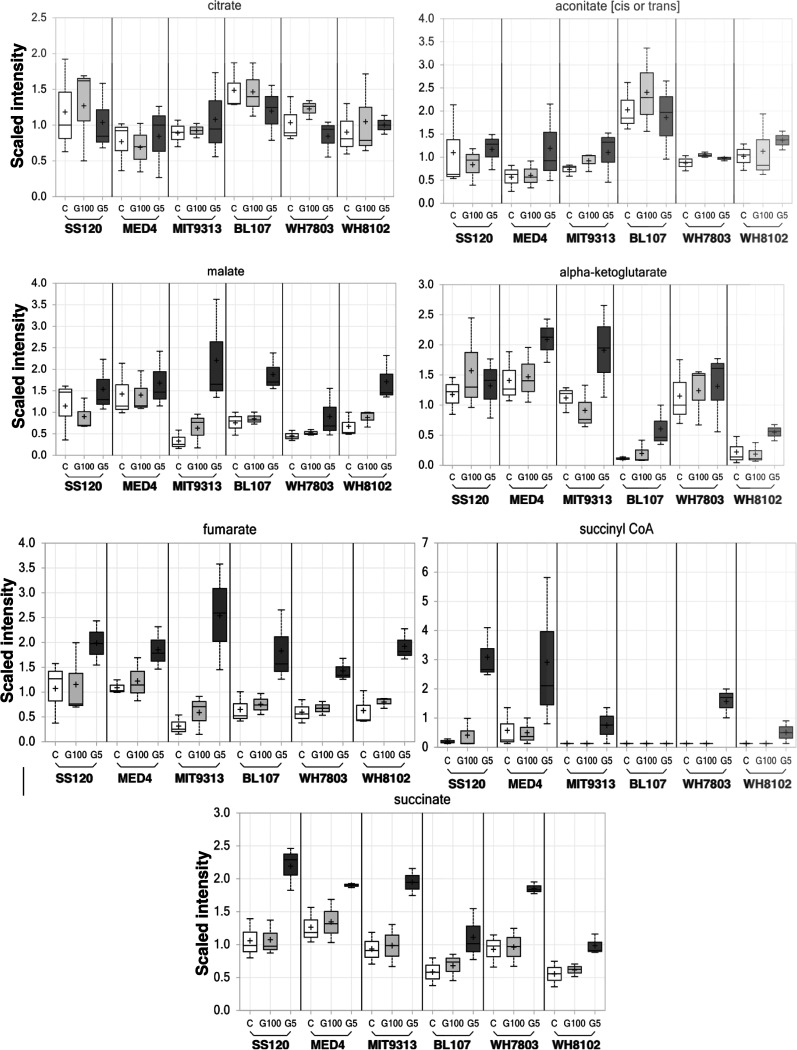
Changes in the concentrations of metabolites related to the tricarboxylic acid cycle. White, control culture; light gray, culture with 100 nM glucose; dark gray, culture with 5 mM glucose.

**FIG 7:**
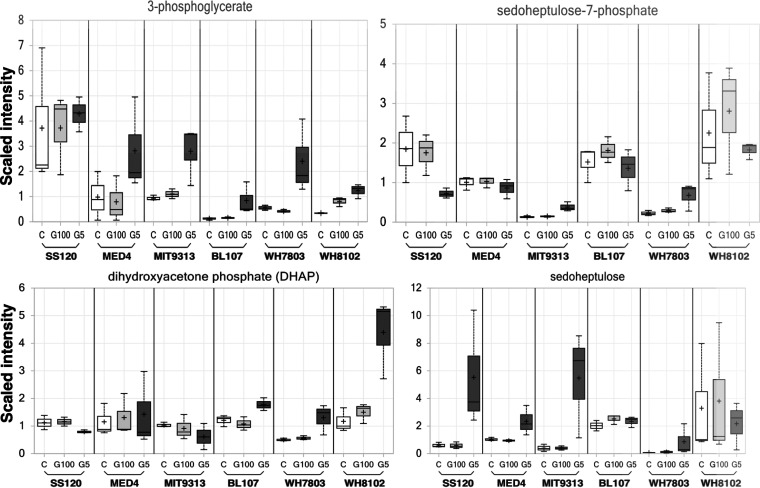
Changes in the concentrations of metabolites related to the Calvin cycle and the pentose phosphate pathway. White, control culture; light gray, culture with 100 nM glucose; dark gray, culture with 5 mM glucose.

Here, we have focused on the study of carbohydrates, especially those that are more related to glucose metabolism in cyanobacterial strains. Specifically, we have given attention to the following metabolic pathways: glycolysis (Fig. 5), TCA cycle (Fig. 6), Calvin cycle, and pentose phosphate pathway (Fig. 7).

Intracellular glucose pools were managed differently in *Prochlorococcus* versus *Synechococcus* strains. It is striking that in *Prochlorococcus* samples the cellular levels of glucose were lowest in the high-glucose treatment cultures; in the *Synechococcus* strains, there was no difference between the treatments, but it was clear that *Synechococcus* sp. BL107 maintained the highest levels of glucose.

Levels of dihydroxyacetone phosphate (also found in the photosynthetic pathways) showed a different effect, i.e., levels were higher in *Synechococcus* samples subjected to 5 mM glucose than in *Prochlorococcus* samples. It may also be significant that the *Synechococcus* strains tended to accumulate trehalose, a disaccharide with strong osmolyte properties (26). Despite these strain differences in the early intermediate pools, all samples showed larger pools of phosphoenolpyruvate, pyruvate, lactate, and acetyl-CoA in samples subjected to high glucose concentrations. This effect was also visible in several intermediates of the TCA cycle (Fig. 6), being higher in the 5 mM glucose samples for succinyl-CoA and all detected derivative compounds in this cycle (succinate, fumarate, and malate).

The effects of glucose availability were also studied in the Calvin cycle and pentose phosphate pathways (Fig. 7). The results showed increases at the highest glucose concentration in 3-phosphoglycerate, except in *Prochlorococcus* sp. SS120. Dihydroxyacetone phosphate showed an increase with the highest glucose concentration in *Synechococcus* samples, but there were no significant differences in the case of *Prochlorococcus*. No significant changes were found in sedoheptulose 7-phosphate pools after glucose addition; the trend was rather linear, or a decrease in samples subjected to 5 mM glucose. On the other hand, the results obtained with sedoheptulose showed that internal glucose pools were differently managed in *Prochlorococcus* versus *Synechococcus*. Sedoheptulose did not show clear increases at the higher glucose concentration in *Synechococcus*. However, *Prochlorococcus* samples showed increased sedoheptulose concentrations at the higher glucose concentration. It should be noted that *Prochlorococcus* SS120 showed a much higher sedoheptulose pool increase than the rest of the strains in the 5 mM glucose-treated samples. It is remarkable that the rest of the metabolites produced in the Calvin cycle and pentose phosphate pathways did not show significant changes at 5 mM glucose in *Prochlorococcus* sp. SS120.

## DISCUSSION

The uptake of organic compounds by marine cyanobacteria has been studied in the last 2 decades by several teams, who have shown these abundant photosynthetic organisms can also take up amino acids, ATP, phosphonates, amino sugars, and sugars, among other compounds (10). However, the specific information about the biological process allowing the uptake and utilization of these compounds is yet very scarce, as is information on their impact on the metabolism of the microbial populations of the oceans.

Glucose uptake was discovered in *Prochlorococcus* (9), where the glucose transporter (GlcH) was identified. We have described a significant degree of diversity in the transporter, in particular for its *K_s_* affinity constant for glucose (14) and for *glcH* expression at different glucose concentrations (15). In early reports, we studied the expression of a number of genes related to glucose metabolism in *Prochlorococccus* sp. strain SS120 (9). We have also studied the effect of nanomolar glucose concentrations added to cultures on the proteome of the same strain (14) and found evidence for glucose utilization but overall a low effect on *Prochlorococcus* metabolism.

Here, we used a more ambitious approach to study the metabolic effects of glucose availability on marine picocyanobacteria; we studied six model strains (three *Synechococcus* and three *Prochlorococcus* strains, selected on the basis of their adaptations to the environment, as described above) and two variables, glucose and light availability. Our goals were to understand how the two key marine picocyanobacterial genera, *Prochlorococcus* and *Synechococcus*, respond to glucose availability in the environment, to shed light on the metabolic strategies for glucose metabolism and possible inter- and intragenus variability, and to assess whether glucose utilization might help these organisms sustain their metabolic needs when growing in the dark.

We decided to study the effect of 100 nM glucose, since that is a concentration relatively similar to that observed in oligotrophic oceans (12). Nevertheless, the proteomic and metabolomic response to 5 mM glucose provided insights in the possible utilization of glucose when available at high concentrations, allowing us to assess the physiological effects of this sugar on *Prochlorococcus* and *Synechococcus*. Other studies analyzing the effects of millimolar concentrations of glucose have shown interesting effects on the photosynthetic apparatus (27) and also on survival under darkness (28) in *Prochlorococcus*. While it is highly improbable to find this glucose concentration in the oceans, its effects are however interesting, since marine picocyanobacteria might be subjected to temporary increases of glucose concentration, which might be assimilated by following the metabolic changes in different pathways described here.

While proteomics (reviewed in reference 29) and metabolomics (30, 31) have been used for a series of studies in cyanobacteria, few of them have been focused on marine picocyanobacteria (32–39). This is, to our knowledge, the first study in which quantitative proteomics have been combined with metabolomics in marine cyanobacteria.

### Proteomics.

**(i) Microbiome changes induced by glucose availability.** Glucose addition induced a significant increase in the levels of heterotrophic bacterial populations in our cyanobacterial cultures (Fig. 1; Fig. S1). This was expected, since the culture medium utilized to grow marine *Synechococcus* and *Prochlorococcus* is devoid of organic compounds and is optimized to support the growth of phototrophic organisms; therefore, addition of a sugar molecule, which is very easy to assimilate, induced a quick response in the heterotrophs’ growth. However, it is remarkable that the increase in the heterotrophic populations upon glucose addition was comparatively low in the cultures of *Synechococcus* sp. strain WH8102 (an oligotrophic strain) and *Prochlorococcus* sp. strain SS120 (which is, as are all *Prochlorococcus* strains, also specialized for growth in oligotrophic environments). This means that the metabolomic concentrations obtained for both strains were mostly the result of metabolism from the cyanobacterial populations and therefore could be considered a significative snapshot of the changes in metabolites synthesized by cyanobacteria upon glucose addition. This fact notwithstanding, it is obvious that definitive conclusions will only be reached when axenic strains for all the cyanobacteria studied here can be obtained and maintained after glucose amendment. As an advantage, the results reported in this study are more representative of the natural conditions than those observed in axenic cultures, since, as indicated in the introduction, *Prochlorococcus* and *Synechococcus* naturally coexist with heterotrophic bacteria in the oceans. Furthermore, there is strong evidence of the importance of interactions with other microorganisms for populations of marine picocyanobacteria (40, 41).

**(ii) Pathways involved in the assimilation of glucose.** Our results confirmed that *Prochlorococcus* and *Synechococcus* respond to glucose availability, showing changes that help in understanding carbon metabolism in these organisms. Together with previous results on these organisms, we have provided evidence that glucose is not only taken up by these microorganisms, but also that it is actually assimilated, inducing significant changes in the proteome and metabolome of the studied strains.

Our results suggest that the main routes involved in glucose utilization in marine picocyanobacteria are the pentose phosphate pathway and the Calvin cycle, and not the Entner-Doudoroff pathway. We observed increases in several enzymes involved in the pentose phosphate pathway (Fig. 2 and 3; Fig. S3), but no signs of direct participation of Entner-Doudoroff pathway-specific enzymes (6-phosphogluconate-dehydratase and 2-keto-3-deoxy-phosphogluconate aldolase) in any of the studied strains. The Entner-Doudoroff pathway had been recently proposed as the prime pathway for glucose utilization in marine picocyanobacteria, particularly in the light (16). Since the pentose phosphate pathway involves enzymes which are common to the Calvin cycle, it was considered that the Entner-Doudoroff pathway would allow utilization of glucose without interfering with Calvin cycle-mediated photosynthesis. However, our results strongly suggest this is not the case, at least under our experimental conditions, so that the oxidative pentose pathway seems to be the preferential pathway for glucose utilization in these organisms, both in the light and in darkness.

Furthermore, our results suggested that there is an activation of the cyclic pentose phosphate pathway, where the enzymes fructose bisphosphate aldolase and triose isomerase work in a direction opposite to glycolysis. This, together with the participation of fructose 1,6-bisphosphatase, allows closure of the cyclic pathway of pentose phosphate, allowing the generation of reducing power from glucose (Fig. 2 and 3; Fig. S3). This means that marine picocyanobacteria can both use glucose as a substrate to make other compounds and also to exploit glucose with energetic purposes. The latter is of special interest for picocyanobacterial populations inhabiting deep waters of the ocean, where there are very low irradiancies available, so that glucose utilization might confer a relevant advantage in order to extend survival, as has been shown by other authors (28, 42).

**(iii) Fermentation.** The increase in the lactate concentration induced by millimolar glucose addition (especially remarkable in cultures of the strain MED4) fits nicely with the upregulation observed for lactate dehydrogenase in *Prochlorococcus* SS120. This points out a putative relevance of the lactic fermentation under those conditions, as proposed in a previous review (43): the presence of lactate dehydrogenase in *Prochlorococcus* sp. strain SS120 allows regeneration of NADH, and thus the continuous transformation of glucose into pyruvate. Furthermore, the expression of the gene encoding lactate dehydrogenase also increases upon glucose addition in *Prochlorococcus* SS120 (9). Consequently, in addition to the possible glucose metabolism through the pentose phosphate or Calvin pathways, strains possessing lactate dehydrogenase could partially degrade glucose to obtain ATP (albeit in lower amounts than from a full glycolysis process) by using the lactic acid fermentation. According to the current version of the Cyanorak database (44; accessed on 27 July 2022), all strains from the LLI, LLII, and LLIII clades from *Prochlorococcus* and some strains from clade CRD1 (subcluster 5.1) of marine *Synechococcus* possess genes encoding lactate dehydrogenase. Consequently, lactic acid fermentation seems to be an important feature in some relevant clades of marine picocyanobacteria.

**(iv) Proteomic changes after glucose addition in the light versus darkness.** The addition of 100 nM glucose did not promote strong changes in the proteomes of *Prochlorococcus* and *Synechococcus*, in agreement with a previous report (14) that found similar results in *Prochlorococcus* sp. strain SS120. The obtained profiles showed similarity between *Synechococcus* strains when a concentration of glucose of 5 mM was added and in dark conditions (regardless of the concentration of glucose); the proteins related to the photosystems, photosynthetic pigments, ATP synthase, and ribosomes showed a strong decrease. The results obtained with *Prochlorococcus* sp. strain SS120 are similar to those found with marine *Synechococcus*, although the decrease of proteins in darkness was considerably more pronounced. This was in agreement with an earlier paper, in which the addition of glucose to *Prochlorococcus* sp. strains SS120 and MED4 subjected to continuous darkness provoked a decrease in *psbA* transcript levels (the gene encoding the D1 protein of photosystem II) (27). This indicates that marine picocyanobacteria change their metabolism, removing the photosynthetic machinery possibly to avoid spending resources on proteins that are not useful under darkness or when a convenient source of carbon and energy (such as glucose) is available.

We observed clear effects of darkness alone on the proteome of the analyzed strains. This was in good agreement with the metabolism of organisms which are primarily autotrophic. A high level of responsiveness to the light conditions was also reported in a study addressing the effects of different light irradiances in several *Synechococcus* strains: more than 50% of abundant proteins showed changes, varying more than 2-fold between the lowest and the highest irradiance. All studied strains, among them one studied here (*Synechococcus* sp. strain WH8102), had decreased phycobilisome protein concentration when irradiance increased (35). Our results showed a general decrease in the abundance of photosynthesis-related proteins in *Prochlorococcus* and *Synechococcus* samples subjected to darkness. However, we found an unexpected result in *Synechococcus* strains: phycobilisome proteins were among the proteins significantly increased upon glucose addition, both in the light and under darkness. This indicated that *Synechococcus* can utilize glucose to strengthen its photosynthetic harvesting system, suggesting that glucose is utilized to fuel primary photosynthetic metabolism. This was in contrast with the reported decrease of *psbA* expression in *Prochlorococcus* (27) and might point to another difference in the adaptive responses of both genera.

The differences in glucose metabolism between *Prochlorococcus* and *Synechococcus* described above (i.e., stronger increases in glucokinase and smaller increases in glucose-6-phosphate dehydrogenase for *Prochlorococcus*) are intriguing and might be related to a different strategy for glucose metabolism in both genera. A possible hypothesis is that the basal levels of glucokinase in *Prochlorococcus* are low (since it is an enzyme consuming ATP), and it therefore requires a strong increase upon glucose addition to allow a good assimilation of the sugar.

It was also striking to observe the differences between *Prochlorococcus* MED4 and SS120 in the dark: the enzymes involved in the oxidative pentose phosphate pathway showed no change upon millimolar glucose addition to MED4 cultures in darkness, while most of them decreased in SS120 under darkness (Fig. S3 versus Fig. 3). Something similar happened in the lower parts of the glucose metabolism pathways, where we also observed remarkable changes in the concentrations of enzymes in SS120, but not in MED4. Therefore, it seems that SS120 is more responsive to glucose addition than MED4. This might be related to the fact that SS120 is a strain adapted to live at depth, and thus glucose utilization might be more beneficial in ecological terms when photosynthesis is limited by low levels of light.

**(v) Kai proteins.** The observed changes in the circadian proteins KaiB and KaiC were especially interesting, since these proteins constitute the core of the circadian clock in cyanobacteria (45). Our results suggested that glucose addition might affect the circadian regulation of metabolism in these microorganisms. Recent results obtained in field studies of *Prochlorococcus* (13) fit nicely with this hypothesis, indicating that glucose addition increases the expression of *kai* genes and it might induce a delay in the circadian rhythms. Since two completely different studies (the current study, with laboratory cultures, and another studying natural populations in the ocean) have found that glucose addition affects the expression of *kai* genes, we believe this is a significant observation and is not caused by any experimental artifact (such as pH variations), but rather is linked to the addition of a sugar to the environment. The adaptive value of such changes, and the physiological mechanism underlying them, remains to be studied.

**(vi) Signal recognition.** Among the proteins showing changes in the proteomic studies, we found a signal recognition particle protein in SS120 which might be involved in glucose sensing at the level of the membrane. Similarly, we were very surprised to see the strong increase in the concentration of PstS in all strains but BL107. While the PstS function in the phosphate transporter has been clearly established in previous studies (46), our results suggested it might somehow be also involved in glucose detection in marine picocyanobacteria. These results will be further assessed in future physiological studies, with the final goal of understanding how glucose is perceived by cyanobacteria and how that detection triggers other metabolic mechanisms allowing these organisms to exploit glucose when available in their environment.

**(vii) Proteomic effects of glucose addition in heterotrophic bacteria.** The dissimilar response in the proteomic results obtained with the heterotrophic bacteria *Marinobacter* and *Alteromonas* subjected to the same nutrient conditions (either control, nanomolar, or millimolar glucose) but growing in light versus darkness was noteworthy. This points to possible metabolic interactions between the heterotrophic bacteria growing in these cultures and the organic exudates produced by *Prochlorococcus* and *Synechococcus*; since marine cyanobacterial cells release organic compounds (exudate) depending on light conditions, the composition of the culture medium can vary in cultures subjected to light versus darkness, thus affecting the metabolism of coexisting heterotrophic bacteria. Interestingly, in earlier studies we observed higher glucose uptake rates in *Prochlorococcus* than in coexisting heterotrophic bacteria in the cultures (9).

### Metabolomics.

It is worth noting that there was generally a good level of correspondence between the results we obtained in the proteomic and metabolomic experiments assessing the effects of glucose availability, indicating there is a concerted response in the physiology of marine *Synechococcus* and *Prochlorococcus* in order to adapt to specific concentrations of glucose. While 100 nM glucose provoked little effect in either the proteome or metabolome (consistent with previous results reported by Muñoz-Marín and coworkers [14]), 5 mM glucose induced important changes which were consistent with the utilization of this sugar by marine picocyanobacteria.

*Synechocystis* sp. strain PCC 6803 can take up and use external glucose as carbon source. Therefore, photoautotrophic and photomixotrophic growth conditions can be directly compared with this cyanobacterium. Transcriptomic and proteomic analyses of glucose effects on *Synechocystis* sp. strain PCC 6803 have been published in recent years (47–50). Metabolomic fingerprints of glucose addition on the primary metabolism in *Synechocystis* sp. strain PCC 6803 have also been published (51, 52). However, this is a cyanobacterium adapted to very different conditions with respect to marine cyanobacteria, with a larger genome and a very complex set of metabolic pathways; therefore, it is difficult to compare results in the two organisms. Despite these limitations, some studies in *Synechocystis* (50) have suggested the direct involvement of the pentose phosphate pathway in glucose assimilation by cyanobacteria, in agreement with our results.

Proteomics and metabolomics reports have revealed that addition of 5 mM glucose under continuous illumination increased the steady-state levels of intermediates of the oxidative pentose phosphate cycle and glycolysis, whereas the intermediates of the Calvin-Benson cycle decreased. Moreover, the storage of carbon in sucrose and glycogen is stimulated by glucose, while TCA cycle metabolites do not seem to be much affected (51, 52). Remarkably, metabolomic analysis also revealed that glucose addition to *Synechocystis* sp. strain PCC 6803 cells in light induced oxidative stress. Metabolites which are characteristic for cells exposed to oxidative stress were identified in the metabolome of cells grown under photoheterotrophic conditions (51).

**(i) Glycolytic intermediates.** Intracellular pools of glycolytic intermediates, including fructose-6-phosphate, 3-phosphoglycerate, 2-phosphoglycerate, and phosphoenolpyruvate, were higher under mixotrophic conditions in *Synechocystis* sp. PCC 6803 in the presence of 5 mM glucose, one of the concentrations used in our study (53). We observed similar results, as all *Prochlorococcus* and *Synechococcus* strains showed increases in phosphoenolpyruvate (PEP), pyruvate, lactate, and acetyl-CoA (Fig. 5). However, dihydroxyacetone phosphate (DHAP) showed two different profiles; *Synechococcus* strains tended to increase DHAP, while *Prochlorococcus* strains tended to decrease or maintain the concentration of this metabolite (Fig. 5). Similar results to those in *Synechococcus* were reported in *Synechocystis* sp. PCC 6803 after 24 h of incubation under photomixotrophic conditions (5 mM glucose and bubbled with air supplemented with 1% CO_2_): DHAP increased, indicating an increase in carbon flow to the oxidative pentose phosphate pathway and glycolysis (53). In cyanobacteria, it was previously proposed that the principal route of glucose catabolism is the oxidative pentose phosphate pathway, although the lower part of the glycolytic pathway is also involved (54).

Moreover, the increment in NADH content under photomixotrophic conditions also shows the enhanced flow of glucose metabolism through the lower parts of the glycolytic pathways (due to the action of glyceraldehyde-3P-dehydrogenase). In other studies, measurement of glucose-6-phosphate and 6-phosphogluconate dehydrogenase activities suggested that the enzymatic activities themselves were not upregulated under photomixotrophic conditions (52). This could indicate that the abundant supply of substrates under mixotrophic conditions drives enhancement of sugar catabolism.

Interestingly, internal glucose pools in *Prochlorococcus* strains tended to decrease, while *Synechococcus* strains maintained stable glucose levels. Another significant change was found with trehalose, which acts differently in *Synechococcus* and *Prochlorococcus*. *Synechococcus* tends to accumulate trehalose, whereas in *Prochlorococcus* it showed a much smaller effect (Fig. 5). It has been reported that Phormidium autumnale sp. LPP_4_ and *Croroococcidiopsis* sp. accumulate trehalose in response to matric water stress. This sugar was accumulated by *Phormidium* in similar concentrations under water stress (55). It is possible that the elevated intracellular salt concentration together with the high concentration of glucose induces the production of sugars like trehalose in *Synechococcus*. In addition, results based on a computational study of the osmoregulation network in response to hyperosmotic stress of *Synechococcus* sp. WH8102 showed that this organism likely uses osmolytes such as trehalose, making it more efficient and adaptable to its changing environment (56).

The amounts of 3-phosphoglycerate, phosphoenolpyruvate, and pyruvate significantly increased upon glucose addition in both *Prochlorococcus* and *Synechococcus* (Fig. 5 and 7), in contrast to the decrease found in *Synechocystis* sp. PCC 6803. The observed decrease in 3-phosphoglycerate, phosphoenolpyruvate, and pyruvate metabolites in *Synechocystis* sp. PCC 6803 under photomixotrophic conditions are probably caused by the repression of the activities of phosphoribulokinase and glyceraldehyde-3-phosphate dehydrogenase (52), and so it seems that regulatory mechanisms differ depending on the type of cyanobacteria.

**(ii) TCA Krebs cycle metabolites.** It would be interesting to complete this work with future studies on photosynthetic activity, since it has been reported in *Synechocystis* sp. PCC 6803 that the decrease in the amount of the Calvin cycle intermediates such as 3PGA under mixotrophic conditions was much larger that under photosynthetic ones (52). It could be that 3PGA scarcely accumulates under photomixotrophic conditions, probably due to the increased metabolic flux to the oxidative pentose phosphate pathway and to the TCA cycle. Among the metabolites of the TCA cycle, the amount of malate, fumarate, succinyl-CoA, and succinate were increased under photomixotrophic conditions (Fig. 6). In cyanobacteria lacking a complete TCA cycle (57), such as the marine picocyanobacteria studied here, succinate is one of the terminal metabolites of the TCA cycle and is a precursor of some biosynthetic reactions. In addition, it is the substrate for succinate dehydrogenase, which is a major component of the cyanobacterial respiratory electron transport chain (58).

**(iii) Calvin cycle and oxidative pentose phosphate metabolites.** The concentration of sedoheptulose-7-phosphate showed a trend to decrease in all *Prochlorococcus* and *Synechococcus* strains in the presence of glucose (Fig. 7), contrary to what was reported in *Synechocystis* sp. PCC 6803, in which they were higher under mixotrophic conditions (53). This suggested that this metabolite acts differently in *Synechocystis* sp. PCC 6803 than in the picocyanobacteria studied in this work. However, sedoheptulose showed a high increment especially in *Prochlorococcus* strains (Fig. 7). 3-Phospoglycerate showed a clear incremental increase in all strains, except in *Prochlorococcus* sp. SS120 (Fig. 7). Late TCA cycle intermediates were higher in all strains relative to controls or low-glucose samples (Fig. 6). These results were in good agreement with those reported by Yoshikawa and coworkers, where intracellular levels of TCA cycle metabolites, such as *cis*-aconitate and succinate, were higher under mixotrophic conditions (53). Decrease of succinate dehydrogenase activity could be the cause of succinate increase under photomixotrophic conditions. Interestingly, the opposite has been reported in *Synechocystis* sp. PCC 6803, where succinate decreased under photomixotrophic conditions (52). Moreover, it is worth noting that the results of ^13^C-metabolic flux analysis suggested that the TCA cycle was less active during autotrophic growth (59). The levels of these TCA cycle metabolites therefore increase under mixotrophic conditions (53).

Some metabolites from the Calvin cycle, i.e., 3-phosphoglycerate and sedoheptulose, were clearly increased in *Prochlorococcus* strains after glucose addition (Fig. 7). However, other compounds did not show any clear change (dehydroxyacetone phosphate, sedoheptulose-7phosphate). This was in contrast with the report of Takahashi and coworkers (52), who observed that carbon flow through the Calvin cycle seemed to decrease under photomixotrophic conditions due to the decrease in the Calvin cycle intermediates and in the photosynthetic activity. This showed that the restriction of photosynthesis is one of the strategies of *Synechocystis* sp. PCC 6803 to adapt to the mixotrophic conditions (52). Our results suggest that *Prochlorococcus* could use a different strategy, where glucose uptake might be used as an additional energy source for photosynthesis, increasing carbon fixation through the Calvin cycle. In *Synechococcus* strains, the changes in Calvin cycle metabolites had little significance, with the exception of dihydroxyacetone phosphate in WH8102 and 3-phosphoglycerate in WH7803, which had important increases after 5 mM glucose addition.

### Conclusion.

Our work suggests that *Prochlorococcus* and *Synechococcus* assimilate glucose by using the pentose phosphate and Calvin cycle pathways (including the cyclic flow to produce reducing power), and apparently not the Entner-Doudoroff pathway, as had been previously proposed. Furthermore, we observed differences in the metabolic strategies utilized by both genera to assimilate glucose, which probably reflected their ecological adaptation, so that metabolism of strains adapted to live in low light (such as *Prochlorococcus* sp. strain SS120) seems to be more responsive to glucose addition than that of high-light-adapted strains (such as MED4). These differences suggest that glucose utilization and, in turn, mixotrophy are part of the standard metabolic strategies in marine picocyanobacteria and have been subjected to fine modulation in the evolutive story of these microorganisms. The actual importance of the utilization of organic compounds by *Prochlorococcus* and *Synechococcus* in the oceans should be further analyzed in order to identify the involved transporters and their contribution to maintenance of picocyanobacterial populations. This would allow researchers to infer the consequences of global warming in the metabolism of these abundant primary producers.

## MATERIALS AND METHODS

### Cyanobacteria culturing and cell collection.

*Prochlorococcus* and marine *Synechococcus* cells were grown in PCR-S11 (60) and ASW media (61), respectively, in a culture room at 24°C. *Prochloloroccus* cells were grown under continuous blue irradiances, 4 μE m^−2^ s^−1^ for low-light-adapted ecotypes (SS120 and MIT9313) and 40 μE m^−2^ s^−1^ for high-light-adapted ecotypes (MED4), using neon Sylvania F18W/154-ST daylight tubes covered with moonlight blue L183 Lee Filters. *Synechococcus* cells were grown under continuous blue light (40 μE m^−2^ s^−1^). When they reached 0.05 units of absorbance (*A*_674_ for *Prochlorococcus*) or 0.1 U of absorbance for *Synechococcus* (*A*_550_ for strains WH7803 and WH8102 and *A*_495_ for BL107), cultures were divided into 6 aliquots and subjected to the different experimental conditions: light, light + 100 nM glucose, light + 5 mM glucose, darkness, darkness + 100 nM glucose, and darkness + 5 mM glucose. For experiments requiring darkness, culture bottles were covered with several layers of aluminum foil.

After 24 h, cells were harvested by centrifugation at 22,000 × *g* for 8 min at 4°C. For metabolomics analysis, cell pellets were frozen in liquid nitrogen after centrifugation and stored at –80°C. For proteomic analysis, pellets were resuspended in 25 mM ammonium bicarbonate and stored at –20°C until used.

It must be noted that *Prochlorococcus* sp. strain MIT9313 cultures stopped growing when we were carrying out the proteomic studies; despite repeated attempts to obtain growing cultures of this strain, we could not achieve that goal. For this reason, proteomic analysis of *Prochlorococcus* sp. strain MIT9313 is not included in this study.

### In-solution trypsin digestion of protein extracts.

After thawing the cell suspensions, total protein concentration was measured using the Bradford assay (62). Samples containing 25 μg of protein were incubated with RapiGest (Waters Corporation) at a final concentration of 0.05% (wt/vol) for 10 min at 80°C. Samples were then reduced with 3 mM dithiothreitol for 10 min at 60°C, followed by alkylation with 9 mM iodoacetamide for 30 min in the dark at room temperature. Finally, trypsin was added (50:1 ratio, protein:enzyme), and samples were incubated overnight at 37°C. To stop the proteolytic reaction and to precipitate the detergent, trifluoroacetic acid (TFA) was added at a final concentration of 0.5% (vol/vol), followed by incubation for 45 min at 37°C. To remove all insoluble material, samples were centrifuged at 13,000 × *g* for 15 min at 4°C.

### Mass spectrometric analysis.

Mass spectrometry (MS) analyses were carried out at the Proteomics Core Facility of the Universidad de Córdoba (Servicio Central de Apoyo a la Investigación). Samples were analyzed as tryptic peptides, resolved by high-resolution liquid chromatography (LC) in an UltiMate 3000 ultrahigh-performance LC system (Thermo Scientific) prior to tandem mass spectrometry (MS/MS) in an Orbitrap Fusion system (Thermo Scientific). Peptide mixtures (500 ng) were trapped onto an Acclaim PepMap RSCL precolumn (300-μm inner diameter, 5 mm long, 5-μm particles; Thermo Scientific) over 3 min, at a flow rate of 5 μL/min in 2% (vol/vol) acetonitrile–0.05% (vol/vol) TFA. Bound peptides were resolved on a C_18_ Acclaim PepMap RSCL nanocolumn (75-μm inner diameter, 50 cm long, 2-μm particles; Thermo Scientific) at 300 nL/min over a 60-min linear gradient from 4% to 40% (vol/vol) acetonitrile in 0.1% (vol/vol) formic acid.

The Orbitrap Fusion system (Thermo Scientific) was operated in data-dependent acquisition mode. The mass range acquisition in full scan mode was *m/z* 400 to 1500 in the Orbitrap at a resolution of 120 K, 4 × 10^5^ AGC ion count target, and 50-ms maximum injection time. Tandem MS was performed by quadrupole isolation at 1.2 Th. The 50 most intense 2 to 5 charged ions were isolated and sequentially fragmented, allowing a 10 ppm tolerance for selected precursors. Precursors selected were dynamically excluded for 15 s. Fragmentation was performed by collision-induced disassociation at a normalized collision energy of 35, and the fragments generated were analyzed in the ion trap.

### Proteomic data analysis.

For proteomic data analysis, label-free quantification was carried out using a Progenesis QI system (Waters). Peak lists obtained were searched against the individual reference proteomes downloaded from UniProt database (June 2021). Reference proteomes contained 2,520 entries for *Synechococcus* sp. strain WH8102, 2,541 entries for *Synechococcus* sp. strain WH7803, 2,501 entries for *Synechococcus* sp. strain BL107, 1,942 entries *Prochlorococcus* strain MED4, and 1,881 entries for *Prochlorococcus* strain SS12. Sequest HT was used as the search engine to obtain protein identifications and imported to Progenesis QI (Waters). Search parameters were set as follows: methionine oxidation as variable modification, carbamidomethylation of cysteine as fixed modification, one trypsin missed cleavage allowed, and an error mass tolerance of 10 ppm for precursors and 0.5 Da for fragment ions. The false-discovery rate was calculated using a decoy database. The abundance of individual proteins was calculated as the ratio of their abundance, calculated for label free, to the sum of the total protein intensity in the sample. For a protein to be considered significantly differentially expressed, it had to be identified and quantified using at least two unique peptides and a *P* value of ≤0.05.

LC-MS/MS data were also processed using Peaks X+ software (BioInformatic Solutions Inc.) to estimate the proportional abundance of different bacteria populations in the cultures. To identify and categorize as many proteins as possible in the samples, we searched our data against an extensive database containing 4,565,004 entries from marine organisms (MarRef v6 database [63]). Search parameters were set as indicated above. Total protein intensity per genus was calculated as the percentage of the sum of the abundance of all proteins belonging to the same genus to total protein intensity. Posttranslational modifications were identified using the PTM Peaks tool with the default parameters.

KEGG pathways visualization was performed using the Pathview package from Bioconductor (64) in R (65). Pathway enrichment analysis was performed using the DAVID functional annotation tool (66, 67). Significant criteria included a *P* value of <0.05, Benjamini score of <0.05, and abundance differences >1.5-fold between groups.

### Metabolomic analysis.

For the metabolomic analysis, the study consisted of 18 triplicate samples, representing 6 cyanobacterial strains: 3 *Prochlorococcus* strains (PCC 9511, SS120, and MIT9313) and 3 *Synechococcus* (WH7803, WH8102, and BL107) under three conditions: light, light + 100 nM glucose, and light + 5 mM glucose. The effect of darkness was not assessed by metabolomic analysis. For each strain, the two glucose conditions were statistically compared with respect to the corresponding control condition (light).

The 54 cyanobacterial cell samples obtained as indicated above were sent to the European headquarters of Metabolon (Potsdam, Germany). Metabolon uses the DiscoveryHD4 platform, which consists of combining multiple mass spectrometry methods and a LIMS system with a large reference library of metabolite standards and a suite of informatics and quality control (QC) software. This allowed the automated identification and quantitation of metabolites. Metabolon performed the determination of the concentrations of the different metabolites, as well as a biostatistical analysis of the results.

Samples were extracted in methanol under vigorous shaking for 2 min (Glen Mills GenoGrinder 2000), followed by centrifugation to recover chemically diverse metabolites. Several recovery standards were added prior to the first step in the extraction process for QC purposes. The resulting extract was divided into five fractions: two for analysis by two separate reverse-phase (RP) UPLC-MS/MS methods using positive ion mode electrospray ionization (ESI), one for analysis by RP UPLC-MS/MS using negative ion mode ESI, one for analysis by hydrophilic interaction chromatography (HILIC) UPLC-MS/MS using negative ion mode ESI, and one reserved for backup. Sample extracts were dried and then reconstituted in solvents compatible to each method. All analyses were performed in an Acquity UPLC system (Waters) coupled to a Q-Exactive mass spectrometer (Thermo Scientific) with a heated electrospray ionization (HESI-II) source. One aliquot was analyzed using acidic positive ion conditions, chromatographically optimized for more hydrophilic compounds. In this method, the extract was eluted from a C_18_ column Waters UPLC BEH column (2.1 by 100 mm, 1.7 μm) using water and methanol as mobile phases and containing 0.05% perfluoropentanoic acid (PFPA) and 0.1% formic acid (FA). A second aliquot was analyzed using acidic positive ion conditions but chromatographically optimized for more hydrophobic compounds. In this method, the extract was gradient eluted from the above-mentioned C_18_ column using methanol, acetonitrile, water, 0.05% PFPA, and 0.01% FA and operated at an overall higher organic content. A third aliquot was analyzed using basic negative ion-optimized conditions with a separate dedicated C_18_ column. The basic extracts were gradient eluted from the column using methanol and water and 6.5 mM ammonium bicarbonate at pH 8. The fourth aliquot was analyzed via negative ionization following elution from a HILIC column (Waters UPLC BEH amide, 2.1 by 150 mm, 1.7 μm) using a gradient consisting of water and acetonitrile with 10 mM ammonium formate, pH 10.8. An Orbitrap mass analyzer was operated in data-dependent acquisition mode at 35,000 mass resolution. The scan range varied slightly between methods but covered approximately 70 to 1,000 *m/z*.

Raw data were extracted, peak identified, and QC processed using Metabolon’s hardware and software. Compounds were identified by comparison to library entries of purified standards or recurrent unknown entities. Metabolon maintains a library based on authenticated standards that contains the retention time or index (RI), mass-to-charge ratio (*m/z*), and chromatographic data (including MS/MS data) on all molecules present in the library. Furthermore, biochemical identifications were based on three criteria: retention index within a narrow RI window of the proposed identification, accurate mass match to the library ±10 ppm, and the MS/MS forward and reverse scores. MS/MS scores were based on a comparison of the ions present in the experimental spectrum to ions present in the library entry spectrum.

Quantification results were scaled by log transformation, and then missing values were imputed with the minimum detected value for that compound. Welch’s two-way *t* tests were used to identify biochemicals that differed significantly between experimental groups. False-discovery rates (*q* values) were estimated to account for multiple comparisons.

### Data availability.

The proteomic data sets utilized in this study are available at ProteomeXchange under accession numbers PXD035483 (*Synechococcus* WH8102), PXD035481 (marine *Synechococcus* WH7803), PXD035395 (*Synechococcus* BL107), PXD035660 (*Prochlorococcus* SS120), and PXD035402 (*Prochlorococcus* MED4). Metabolomics data have been deposited in the EMBL-EBI MetaboLights database with identifier MTBLS5462.

## References

[1] Visintini N, Martiny AC, Flombaum P. 2021. *Prochlorococcus*, *Synechococcus*, and picoeukaryotic phytoplankton abundances in the global ocean. Limnol Oceanogr Lett 6:207–215. doi:10.1002/lol2.10188.

[2] Flombaum P, Martiny AC. 2021. Diverse but uncertain responses of picophytoplankton lineages to future climate change. Limnol Oceanogr 66:4171–4181. doi:10.1002/lno.11951.

[3] Flombaum P, Gallegos JL, Gordillo RA, Rincón J, Zabala LL, Jiao N, Karl DM, Li WKW, Lomas MW, Veneziano D, Vera CS, Vrugt JA, Martiny AC. 2013. Present and future global distributions of the marine cyanobacteria *Prochlorococcus* and *Synechococcus*. Proc Natl Acad Sci USA 110:9824–9829. doi:10.1073/pnas.1307701110. 23703908 PMC3683724

[4] Dufresne A, Salanoubat M, Partensky F, Artiguenave F, Axmann I, Barbe V, Duprat S, Galperin M, Koonin E, Le Gall F, Makarova K, Ostrowski M, Oztas S, Robert C, Rogozin I, Scanlan D, Tandeau de Marsac N, Weissenbach J, Wincker P, Wolf Y, Hess W. 2003. Genome sequence of the cyanobacterium *Prochlorococcus marinus* SS120, a nearly minimal oxyphototrophic genome. Proc Natl Acad Sci USA 100:10020–10025. doi:10.1073/pnas.1733211100. 12917486 PMC187748

[5] Zubkov M, Fuchs B, Tarran G, Burkill P, Amann R. 2003. High rate of uptake of organic nitrogen compounds by *Prochlorococcus* cyanobacteria as a key to their dominance in oligotrophic oceanic waters. Appl Environ Microbiol 69:1299–1304. doi:10.1128/AEM.69.2.1299-1304.2003. 12571062 PMC143617

[6] Sosa OA, Casey JR, Karl DM. 2019. Methylphosphonate oxidation in *Prochlorococcus* strain MIT9301 supports phosphate acquisition, formate excretion, and carbon assimilation into purines. Appl Environ Microbiol 85:e00289-19. doi:10.1128/AEM.00289-19. 31028025 PMC6581173

[7] Duhamel S, Van Wambeke F, Lefevre D, Benavides M, Bonnet S. 2018. Mixotrophic metabolism by natural communities of unicellular cyanobacteria in the western tropical South Pacific Ocean. Environ Microbiol 20:2743–2756. doi:10.1111/1462-2920.14111. 29573372

[8] Vila-Costa M, Simo R, Harada H, Gasol J, Slezak D, Kiene R. 2006. Dimethylsulfoniopropionate uptake by marine phytoplankton. Science 314:652–654. doi:10.1126/science.1131043. 17068265

[9] Gómez-Baena G, López-Lozano A, Gil-Martínez J, Lucena J, Diez J, Candau P, García-Fernández J. 2008. Glucose uptake and its effect on gene expression in *Prochlorococcus*. PLoS One 3:e3416. doi:10.1371/journal.pone.0003416. 18941506 PMC2565063

[10] Muñoz-Marín MC, Gómez-Baena G, López-Lozano FA, Moreno-Cabezuelo JA, Díez J, García-Fernández JM. 2020. Mixotrophy in marine picocyanobacteria: use of organic compounds by *Prochlorococcus* and *Synechococcus*. ISME J 14:1065–1073. doi:10.1038/s41396-020-0603-9. 32034281 PMC7174365

[11] Wu Z, Aharonovich D, Roth-Rosenberg D, Weissberg O, Luzzatto-Knaan T, Vogts A, Zoccarato L, Eigenmann F, Grossart H-P, Voss M, Follows M, Sher D. 2022. Single-cell measurements and modelling reveal substantial organic carbon acquisition by *Prochlorococcus*. Nat Microbiol 7:2068–2077. doi:10.1038/s41564-022-01250-5. 36329198 PMC9712107

[12] Muñoz-Marín MC, Luque I, Zubkov MV, Hill PG, Diez J, García-Fernández JM. 2013. *Prochlorococcus* can use the Pro1404 transporter to take up glucose at nanomolar concentrations in the Atlantic Ocean. Proc Natl Acad Sci USA 110:8597–8602. doi:10.1073/pnas.1221775110. 23569224 PMC3666668

[13] Muñoz-Marín MC, Duhamel S, Björkman KMMJ, Diez J, Karl DM, García-Fernández JM. 2022. Differential timing for glucose assimilation in *Prochlorococcus* and coexistent microbial populations in the North Pacific subtropical gyre. Microbiol Spectr 10:e0246622. doi:10.1128/spectrum.02466-22. 36098532 PMC9602893

[14] Muñoz-Marín MC, Gómez-Baena G, Diez J, Beynon RJ, González-Ballester D, Zubkov MV, García-Fernández JM. 2017. Glucose uptake in *Prochlorococcus*: diversity of kinetics and effects on the metabolism. Front Microbiol 8:327. doi:10.3389/fmicb.2017.00327. 28337178 PMC5340979

[15] Moreno-Cabezuelo JA, López-Lozano A, Díez J, García-Fernández JM. 2019. Differential expression of the glucose transporter gene *glcH* in response to glucose and light in marine picocyanobacteria. PeerJ 6:e6248. doi:10.7717/peerj.6248. 30648008 PMC6330958

[16] Chen X, Schreiber K, Appel J, Makowka A, Fahnrich B, Roettger M, Hajirezaei MR, Sonnichsen FD, Schonheit P, Martin WF, Gutekunst K. 2016. The Entner-Doudoroff pathway is an overlooked glycolytic route in cyanobacteria and plants. Proc Natl Acad Sci USA 113:5441–5446. doi:10.1073/pnas.1521916113. 27114545 PMC4868481

[17] Noh Y, Lee H, Kim M, Hong SJ, Lee H, Kim DM, Cho BK, Lee CG, Choi HK. 2021. Enhanced production of photosynthetic pigments and various metabolites and lipids in the cyanobacteria *Synechocystis* sp. PCC 7338 culture in the presence of exogenous glucose. Biomolecules 11:214. doi:10.3390/biom11020214. 33546462 PMC7913732

[18] Partensky F, Hoepffner N, Li W, Ulloa O, Vaulot D. 1993. Photoacclimation of *Prochlorococcus* sp (Prochlorophyta) strains isolated from the North Atlantic and the Mediterranean sea. Plant Physiol 101:285–296. doi:10.1104/pp.101.1.285. 12231684 PMC158675

[19] Rocap G, Larimer F, Lamerdin J, Malfatti S, Chain P, Ahlgren N, Arellano A, Coleman M, Hauser L, Hess W, Johnson Z, Land M, Lindell D, Post A, Regala W, Shah M, Shaw S, Steglich C, Sullivan M, Ting C, Tolonen A, Webb E, Zinser E, Chisholm S. 2003. Genome divergence in two *Prochlorococcus* ecotypes reflects oceanic niche differentiation. Nature 424:1042–1047. doi:10.1038/nature01947. 12917642

[20] Rocap G, Distel D, Waterbury J, Chisholm S. 2002. Resolution of *Prochlorococcus* and *Synechococcus* ecotypes by using 16S-23S ribosomal DNA internal transcribed spacer sequences. Appl Environ Microbiol 68:1180–1191. doi:10.1128/AEM.68.3.1180-1191.2002. 11872466 PMC123739

[21] Chisholm S, Frankel S, Goericke R, Olson R, Palenik B, Waterbury J, Westjohnsrud L, Zettler E. 1992. *Prochlorococcus marinus* nov. gen. nov. sp.: an oxyphototrophic marine prokaryote containing divinyl chlorophyll *a* and *b*. Arch Microbiol 157:297–300. doi:10.1007/BF00245165.

[22] Brahamsha B. 1996. An abundant cell-surface polypeptide is required for swimming by the nonflagellated marine cyanobacterium *Synechococcus*. Proc Natl Acad Sci USA 93:6504–6509. doi:10.1073/pnas.93.13.6504. 8692845 PMC39053

[23] Waterbury J, Watson S, Valois F, Franks D. 1986. Biological and ecological characterization of the marine unicellular cyanobacterium *Synechococcus*. Can J Fish Aquat Sci 214:71–120.

[24] Dufresne A, Ostrowski M, Scanlan D, Garczarek L, Mazard S, Palenik B, Paulsen I, Tandeau de Marsac N, Wincker P, Dossat C, Ferriera S, Johnson J, Post A, Hess W, Partensky F. 2008. Unraveling the genomic mosaic of a ubiquitous genus of marine cyanobacteria. Genome Biol 9:R90. doi:10.1186/gb-2008-9-5-r90. 18507822 PMC2441476

[25] Tomita J, Nakajima M, Kondo T, Iwasaki H. 2005. No transcription-translation feedback in circadian rhythm of KaiC phosphorylation. Science 307:251–254. doi:10.1126/science.1102540. 15550625

[26] Scanlan DJ, Ostrowski M, Mazard S, Dufresne A, Garczarek L, Hess WR, Post AF, Hagemann M, Paulsen I, Partensky F. 2009. Ecological genomics of marine picocyanobacteria. Microbiol Mol Biol Rev 73:249–299. doi:10.1128/MMBR.00035-08. 19487728 PMC2698417

[27] García-Fernández J, Hess W, Houmard J, Partensky F. 1998. Expression of the *psbA* gene in the marine oxyphotobacteria *Prochlorococcus* spp. Arch Biochem Biophys 359:17–23. doi:10.1006/abbi.1998.0862. 9799555

[28] Coe A, Ghizzoni J, LeGault K, Biller S, Roggensack SE, Chisholm SW. 2016. Survival of *Prochlorococcus* in extended darkness. Limnol Oceanogr 61:1375–1388. doi:10.1002/lno.10302.

[29] Battchikova N, Muth-Pawlak D, Aro EM. 2018. Proteomics of cyanobacteria: current horizons. Curr Opin Biotechnol 54:65–71. doi:10.1016/j.copbio.2018.02.012. 29499477

[30] Wang Y, Chen L, Zhang W. 2016. Proteomic and metabolomic analyses reveal metabolic responses to 3-hydroxypropionic acid synthesized internally in cyanobacterium *Synechocystis* sp. PCC 6803. Biotechnol Biofuels 9:209. doi:10.1186/s13068-016-0627-6. 27757169 PMC5053081

[31] Schwarz D, Orf I, Kopka J, Hagemann M. 2013. Recent applications of metabolomics toward cyanobacteria. Metabolites 3:72–100. doi:10.3390/metabo3010072. 24957891 PMC3901253

[32] Christie-Oleza JA, Armengaud J. 2015. Proteomics of the *Roseobacter* clade, a window to the marine microbiology landscape. Proteomics 15:3928–3942. 26415894 10.1002/pmic.201500222

[33] Christie-Oleza JA, Scanlan DJ, Armengaud J. 2015. “You produce while I clean up,” a strategy revealed by exoproteomics during *Synechococcus*-*Roseobacter* interactions. Proteomics 15:3454–3462. doi:10.1002/pmic.201400562. 25728650 PMC4949626

[34] Kaur A, Hernandez-Fernaud JR, Aguilo-Ferretjans MD, Wellington EM, Christie-Oleza JA. 2018. 100 days of marine *Synechococcus-Ruegeria* pomeroyi interaction: a detailed analysis of the exoproteome. Environ Microbiol 20:785–799. doi:10.1111/1462-2920.14012. 29194907 PMC5839243

[35] Mackey KRM, Post AF, McIlvin MR, Saito MA. 2017. Physiological and proteomic characterization of light adaptations in marine *Synechococcus*. Environ Microbiol 19:2348–2365. doi:10.1111/1462-2920.13744. 28371229

[36] Saito M, McIlvin M, Moran D, Goepfert T, DiTullio G, Post A, Lamborg C. 2014. Multiple nutrient stresses at intersecting Pacific Ocean biomes detected by protein biomarkers. Science 345:1173–1177. doi:10.1126/science.1256450. 25190794

[37] Mackey KR, Post AF, McIlvin MR, Cutter GA, John SG, Saito MA. 2015. Divergent responses of Atlantic coastal and oceanic *Synechococcus* to iron limitation. Proc Natl Acad Sci USA 112:9944–9949. doi:10.1073/pnas.1509448112. 26216989 PMC4538626

[38] Christie-Oleza JA, Armengaud J, Guerin P, Scanlan DJ. 2015. Functional distinctness in the exoproteomes of marine *Synechococcus*. Environ Microbiol 17:3781–3794. doi:10.1111/1462-2920.12822. 25727668 PMC4949707

[39] Casey JR, Boiteau RM, Engqvist MKM, Finkel ZV, Li G, Liefer J, Muller CL, Munoz N, Follows MJ. 2022. Basin-scale biogeography of marine phytoplankton reflects cellular-scale optimization of metabolism and physiology. Sci Adv 8:eabl4930. doi:10.1126/sciadv.abl4930. 35061539 PMC8782455

[40] Christie-Oleza JA, Sousoni D, Lloyd M, Armengaud J, Scanlan DJ. 2017. Nutrient recycling facilitates long-term stability of marine microbial phototroph-heterotroph interactions. Nat Microbiol 2:17100. doi:10.1038/nmicrobiol.2017.100. 28650444 PMC5495174

[41] Follett CL, Dutkiewicz S, Ribalet F, Zakem E, Caron D, Armbrust EV, Follows MJ. 2022. Trophic interactions with heterotrophic bacteria limit the range of *Prochlorococcus*. Proc Natl Acad Sci USA 119:e2110993118. doi:10.1073/pnas.2110993118. 34983874 PMC8764666

[42] Coe A, Biller SJ, Thomas E, Boulias K, Bliem C, Arellano A, Dooley K, Rasmussen AN, LeGault K, ÓKeefe TJ, Stover S, Greer EL, Chrisholm SW. 2021. Coping with darkness: the adaptive response of marine picocyanobacterial to repeat light energy deprivation. Limnol Oceanogr 66:3300–3312. doi:10.1002/lno.11880. 34690365 PMC8518828

[43] García-Fernández J, Diez J. 2004. Adaptive mechanisms of the nitrogen and carbon assimilatory pathways in the marine cyanobacteria *Prochlorococcus*. Res Microbiol 155:795–802. doi:10.1016/j.resmic.2004.06.009. 15567272

[44] Garczarek L, Guyett U, Doré H, Farrantt GK, Hoebeke M, Brillet-Guéguen L, Bisch A, Ferrieux M, Siltanen J, Corre E, Le Corguillé G, Ratin M, Pitt FD, Ostrowski M, Conan M, Siegel A, Labadie K, Aury JM, Wincker P, Scanlan DJ, Partensky F. 2021. Cyanorak v2.1: a scalable information system dedicated to the visualization and expert curation of marine and brackish picocyanobacteria genomes. Nucleic Acids Res 49:D667–D676. doi:10.1093/nar/gkaa958. 33125079 PMC7779031

[45] Chavan AG, Swan JA, Heisler J, Sancar C, Ernst DC, Fang M, Palacios JG, Spangler RK, Bagshaw CR, Tripathi S, Crosby P, Golden SS, Partch CL, LiWang A. 2021. Reconstitution of an intact clock reveals mechanisms of circadian timekeeping. Science 374:eabd4453. doi:10.1126/science.abd4453. 34618577 PMC8686788

[46] Scanlan DJ, Mann NH, Carr NG. 1993. The response of the picoplanktonic marine cyanobacterium *Synechococcus* species WH7803 to phosphate starvation involves a protein homologous to the periplasmic phosphate-binding protein of *Escherichia coli*. Mol Microbiol 10:181–191. doi:10.1111/j.1365-2958.1993.tb00914.x. 7968514

[47] Yang C, Qiang H, Shimizu K. 2002. Metabolic flux analysis in *Synechocystis* using isotope distribution from 13C-labeled glucose. Metab Eng 4:202–216. doi:10.1006/mben.2002.0226. 12616690

[48] Kahlon S, Beeri K, Ohkawa H, Hihara Y, Murik O, Suzuki I, Ogawa T, Kaplan A. 2006. A putative sensor kinase, Hik31, is involved in the response of *Synechocystis* sp. strain PCC 6803 to the presence of glucose. Microbiology 152:647–655. doi:10.1099/mic.0.28510-0. 16514145

[49] Herranen M, Battchikova N, Zhang P, Graf A, Sirpiö S, Paakkarinen V, Aro E. 2004. Towards functional proteomics of membrane protein complexes in *Synechocystis* sp. PCC 6803. Plant Physiol 134:470–481. doi:10.1104/pp.103.032326. 14730074 PMC316326

[50] Ge HT, Fang LF, Huang XH, Wang JL, Chen WY, Zhang YY, Wang XR, Sui N, Xu W, He QF, Wang YC. 2018. Activation of the oxidative pentose phosphate pathway is critical for photomixotrophic growth of a hik33-deletion mutant of *Synechocystis* sp. PCC 6803. Proteomics 18:e1800046. doi:10.1002/pmic.201800046. 30194912

[51] Narainsamy K, Cassier-Chauvat C, Junot C, Chauvat F. 2013. High performance analysis of the cyanobacterial metabolism via liquid chromatography coupled to a LTQ-Orbitrap mass spectrometer: evidence that glucose reprograms the whole carbon metabolism and triggers oxidative stress. Metabolomics 9:21–32. doi:10.1007/s11306-011-0382-4.

[52] Takahashi H, Uchimiya H, Hihara Y. 2008. Difference in metabolite levels between photoautotrophic and photomixotrophic cultures of *Synechocystis* sp PCC 6803 examined by capillary electrophoresis electrospray ionization mass spectrometry. J Exp Bot 59:3009–3018. doi:10.1093/jxb/ern157. 18611912 PMC2504344

[53] Yoshikawa K, Hirasawa T, Ogawa K, Hidaka Y, Nakajima T, Furusawa C, Shimizu H. 2013. Integrated transcriptomic and metabolomic analysis of the central metabolism of *Synechocystis* sp PCC 6803 under different trophic conditions. Biotechnol J 8:571–580. doi:10.1002/biot.201200235. 23495147

[54] Pelroy R, Rippka R, Stanier R. 1972. Metabolism of glucose by unicellular blue-green algae. Arch Microbiol 87:303–322. 10.1007/BF00409131 4629415

[55] Hershkovitz N, Oren A, Cohen Y. 1991. Accumulation of trehalose and sucrose in cyanobacteria exposed to matric water-stress. Appl Environ Microbiol 57:645–648. doi:10.1128/aem.57.3.645-648.1991. 16348431 PMC182773

[56] Mao XZ, Olman V, Stuart R, Paulsen IT, Palenik B, Xu Y. 2010. Computational prediction of the osmoregulation network in *Synechococcus* sp. WH8102. BMC Genomics 11:291. doi:10.1186/1471-2164-11-291. 20459751 PMC2874817

[57] Zhang S, Bryant DA. 2011. The tricarboxylic acid cycle in cyanobacteria. Science 334:1551–1553. doi:10.1126/science.1210858. 22174252

[58] Cooley J, Vermaas W. 2001. Succinate dehydrogenase and other respiratory pathways in thylakoid membranes of *Synechocystis* sp. strain PCC 6803: capacity comparisons and physiological function. J Bacteriol 183:4251–4258. doi:10.1128/JB.183.14.4251-4258.2001. 11418566 PMC95315

[59] Young JD, Shastri AA, Stephanopoulos G, Morgan JA. 2011. Mapping photoautotrophic metabolism with isotopically nonstationary C-13 flux analysis. Metab Eng 13:656–665. doi:10.1016/j.ymben.2011.08.002. 21907300 PMC3210925

[60] El Alaoui S, Diez J, Humanes L, Toribio F, Partensky F, García-Fernández J. 2001. *In vivo* regulation of glutamine synthetase activity in the marine chlorophyll *b*-containing cyanobacterium *Prochlorococcus* sp. strain PCC 9511 (Oxyphotobacteria). Appl Environ Microbiol 67:2202–2207. doi:10.1128/AEM.67.5.2202-2207.2001. 11319101 PMC92856

[61] Moore L, Coe A, Zinser E, Saito M, Sullivan M, Lindell D, Frois-Moniz K, Waterbury J, Chisholm S. 2007. Culturing the marine cyanobacterium *Prochlorococcus*. Limnol Oceanogr Methods 5:353–362. doi:10.4319/lom.2007.5.353.

[62] Bradford M. 1976. A rapid and sensitive method for the quantitation of microgram quantities of protein utilizing the principle of protein-dye binding. Anal Biochem 72:248–254. doi:10.1006/abio.1976.9999. 942051

[63] Klemetsen T, Raknes IA, Fu J, Agafonov A, Balasundaram SV, Tartari G, Robertsen E, Willassen NP. 2018. The MAR databases: development and implementation of databases specific for marine metagenomics. Nucleic Acids Res 46:D692–D699. doi:10.1093/nar/gkx1036. 29106641 PMC5753341

[64] Luo W, Brouwer C. 2013. Pathview: an R/Bioconductor package for pathway-based data integration and visualization. Bioinformatics 29:1830–1831. doi:10.1093/bioinformatics/btt285. 23740750 PMC3702256

[65] R Core Team. 2016. R: a language and environment for statistical computing. R Foundation for Statistical Computing, Vienna, Austria.

[66] Sherman BT, Hao M, Qiu J, Jiao X, Baseler MW, Lane HC, Imamichi T, Chang W. 2022. DAVID: a web server for functional enrichment analysis and functional annotation of gene lists (2021 update). Nucleic Acids Res 50:W216–W221. doi:10.1093/nar/gkac194. 35325185 PMC9252805

[67] Huang da W, Sherman BT, Lempicki RA. 2009. Systematic and integrative analysis of large gene lists using DAVID bioinformatics resources. Nat Protoc 4:44–57. doi:10.1038/nprot.2008.211. 19131956

