# Supplementary material for: Integrated Proteomic and Metabolomic Analyses Show Differential Effects of Glucose Availability in Marine Synechococcus and Prochlorococcus
Source: Microbiol Spectr. 2023 Feb 1;11(2):e03275-22. doi: 10.1128/spectrum.03275-22 (PMC10100731; doi:10.1128/spectrum.03275-22)
Supplement: Supplemental file 5 — Fig. S1 to S3 and legends for Tables S1 to S4. Download spectrum.03275-22-s0005.pdf, PDF file, 3.0 MB [file spectrum.03275-22-s0005.pdf]

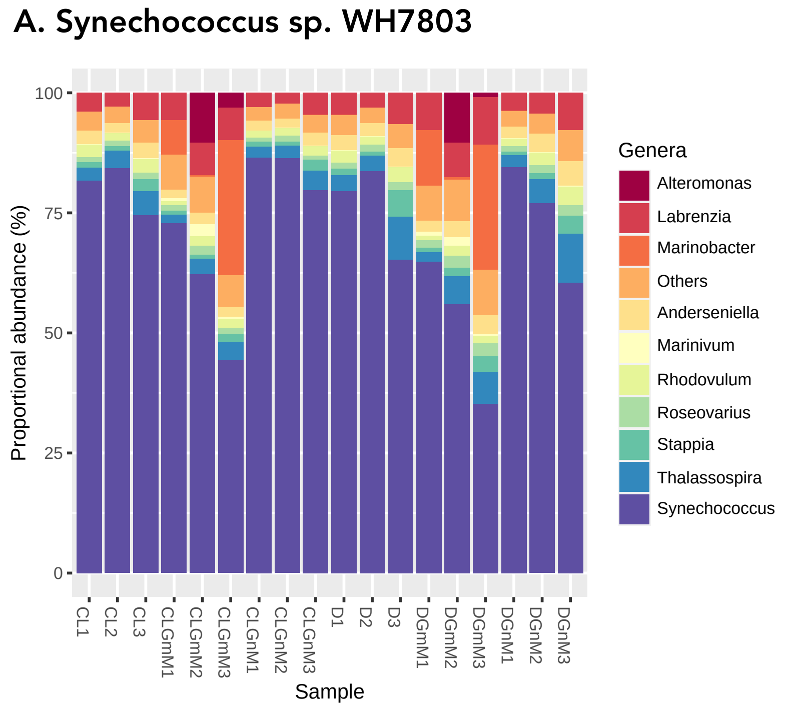

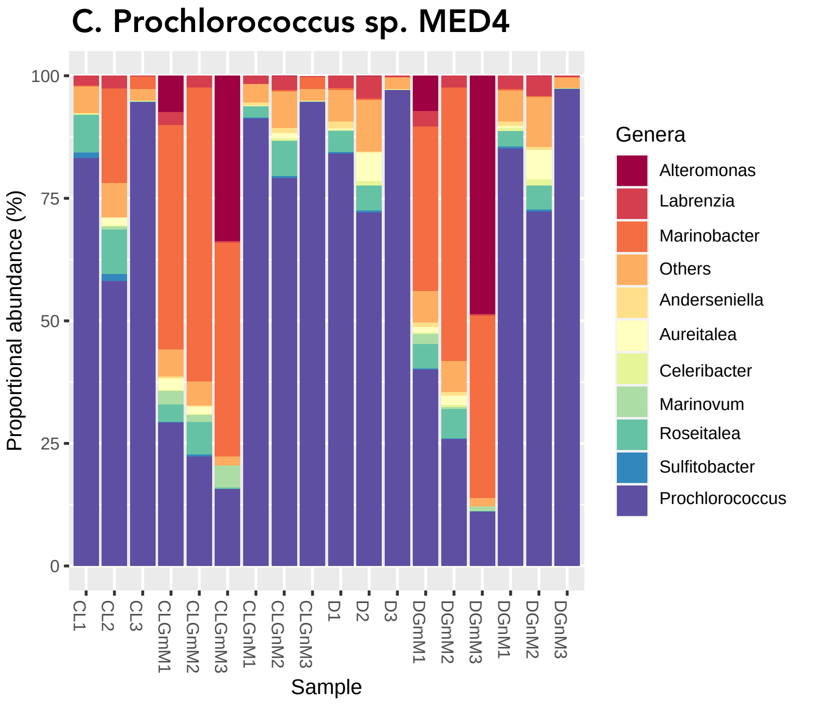

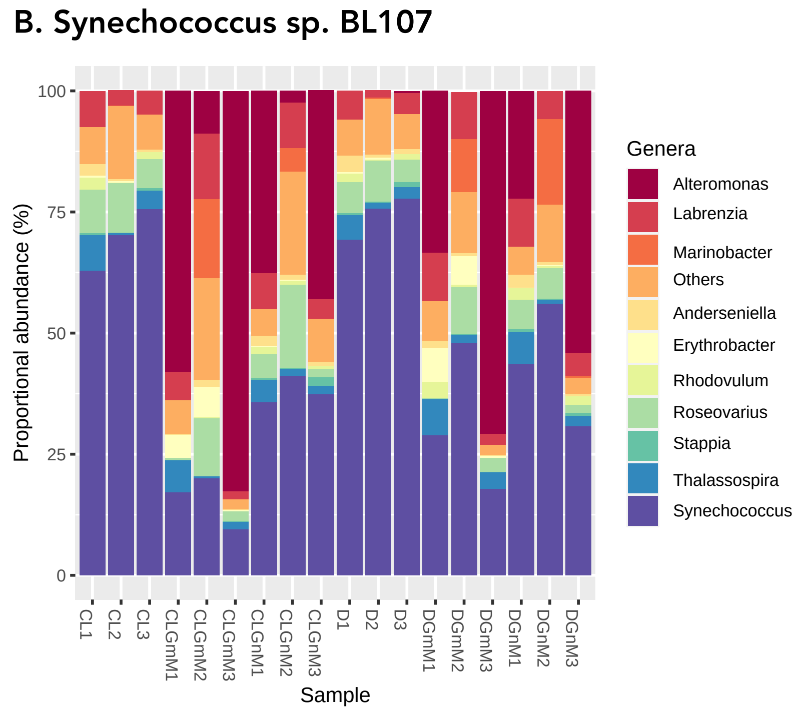

Supplementary Figure 1: Proportional abundance of different genera in cultures from *Synechococcus* and *Prochlorococcus*. Panel A shows results from *Synechococcus* sp WH7803 cultures. Panel B shows the results from *Synechococcus* sp BL107 cultures. Panel C showns the results from *Prochlorococcus* sp MED4 cultures. Proportional abundance was calculated based on total protein intensity per genera obtained by relative label-free abundance using Peaks X (Bioinformatics sol.) divided by total protein intensity per sample. Each column represent a sample. CL=control light, D=Darkess, GmM= glucose addition 5 mM, GnM= glucose addition 100 nM. Three biological replicates were prepared per condition (1,2,3).

WH8102

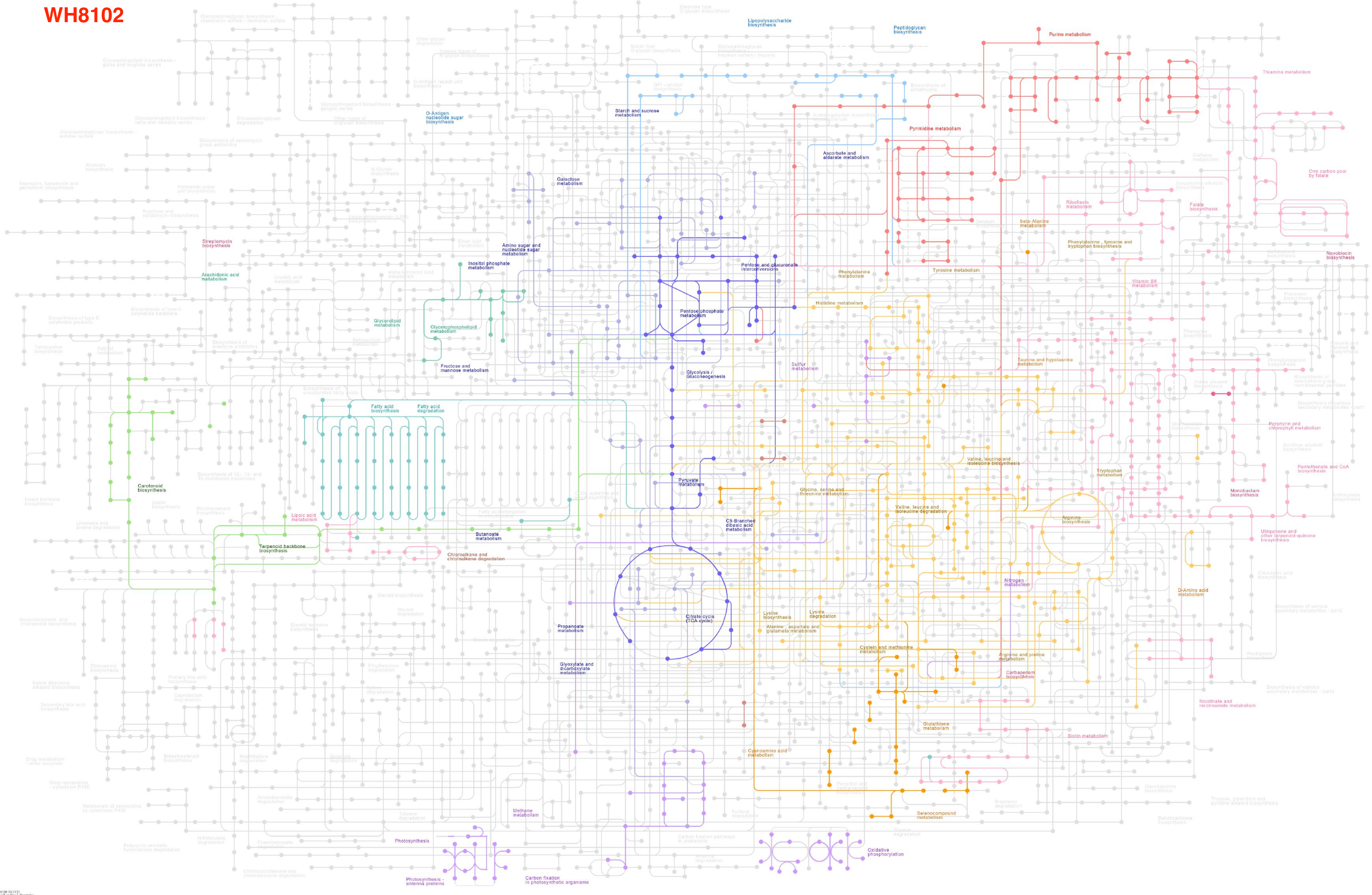

01100 10/13/21
(c) Kanehisa Laboratories

WH7803

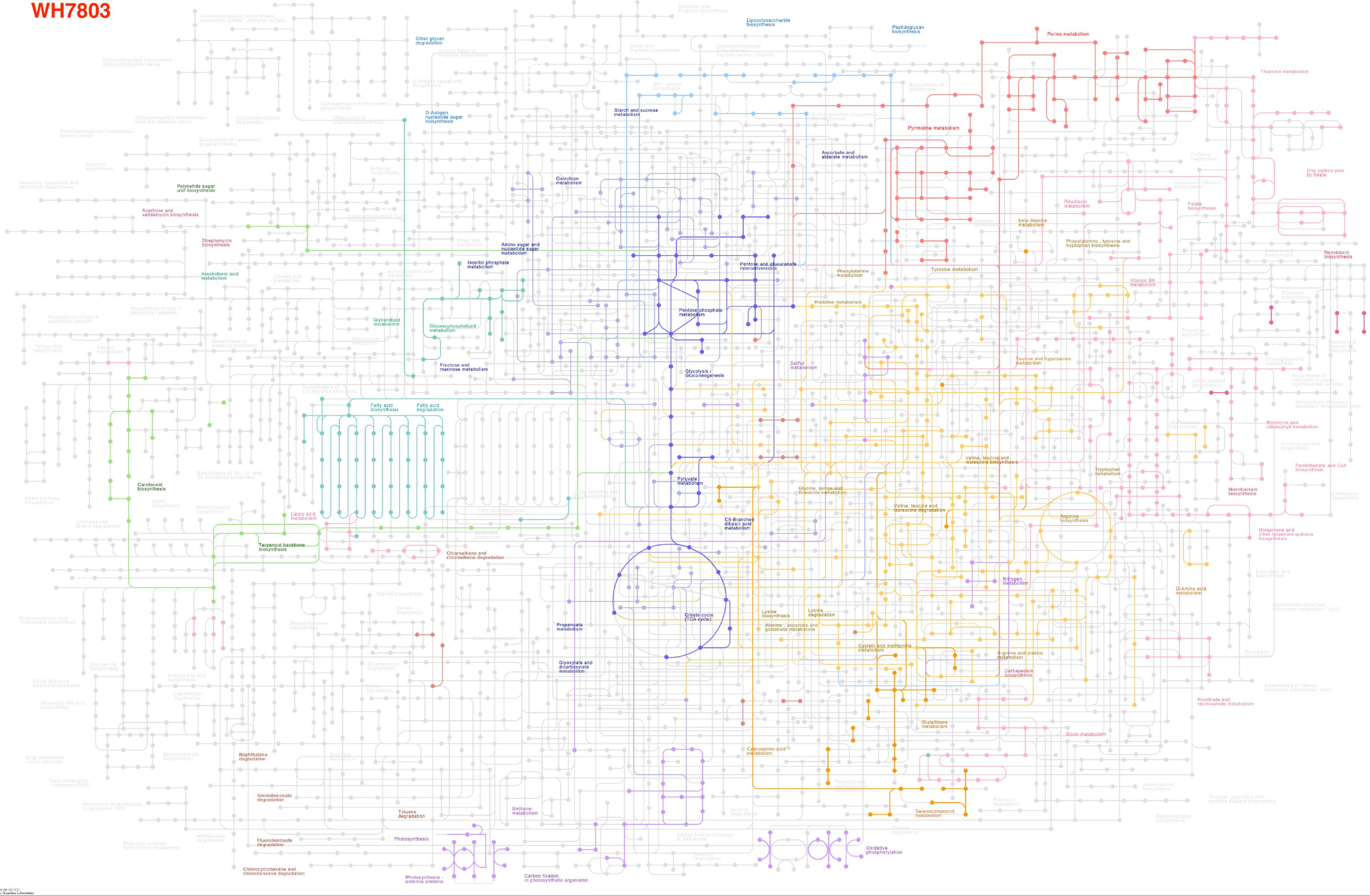

01100 10/13/21
(c) Kanehisa Laboratories

SS120

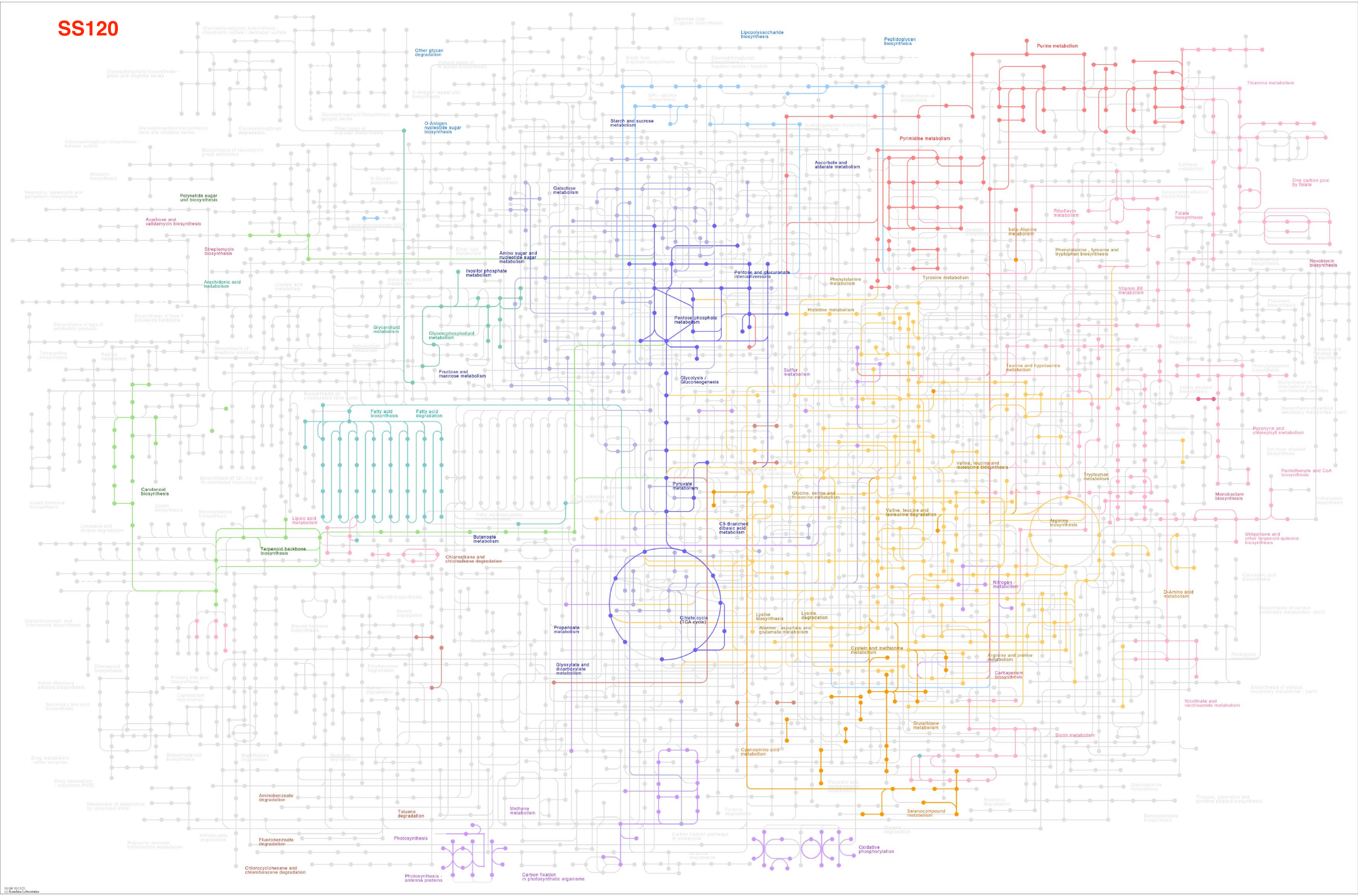

Lipopolysaccharide biosynthesis
Peptidoglycan biosynthesis
Purine metabolism
Other glycan degradation
Thiamine metabolism
O-Antigen nucleotide sugar biosynthesis
Starch and sucrose metabolism
Pyrimidine metabolism
Ascorbate and aldarate metabolism
One carbon pool by folate
Polyketide sugar unit biosynthesis
Galactose metabolism
Riboflavin metabolism
Folate biosynthesis
Acarbose and validamycin biosynthesis
beta-Alanine metabolism
Phenylalanine , tyrosine and tryptophan biosynthesis
Streptomycin biosynthesis
Amino sugar and nucleotide sugar metabolism
Novobiocin biosynthesis
Inositol phosphate metabolism
Pentose and glucuronate interconversions
Tyrosine metabolism
Arachidonic acid metabolism
Phenylalanine metabolism
Vitamin B6 metabolism
Histidine metabolism
Glycerolipid metabolism
Glycerophospholipid metabolism
Pentose phosphate metabolism
Fructose and mannose metabolism
Sulfur metabolism
Taurine and hypotaurine metabolism
Glycolysis / Gluconeogenesis
Fatty acid biosynthesis
Fatty acid degradation
Porphyrin and chlorophyll metabolism
Valine, leucine and isoleucine biosynthesis
Pantothenate and CoA biosynthesis
Tryptophan metabolism
Pyruvate metabolism
Carotenoid biosynthesis
Glycine, serine and threonine metabolism
Monobactam biosynthesis
Valine, leucine and isoleucine degradation
Lipoic acid metabolism
C5-Branched dibasic acid metabolism
Arginine biosynthesis
Ubiquinone and other terpenoid-quinone biosynthesis
Butanoate metabolism
Terpenoid backbone biosynthesis
Chloroalkane and chloroalkene degradation
Nitrogen metabolism
D-Amino acid metabolism
Citrate cycle (TCA cycle)
Propanoate metabolism
Lysine biosynthesis
Lysine degradation
Alanine , aspartate and glutamate metabolism
Cystein and methionine metabolism
Arginine and proline metabolism
Glyoxylate and dicarboxylate metabolism
Carbapenem biosynthesis
Nicotinate and nicotinamide metabolism
Glutathione metabolism
Biotin metabolism
Cyanoamino acid metabolism
Aminobenzoate degradation
Toluene degradation
Methane metabolism
Selenocompound metabolism
Fluorobenzoate degradation
Photosynthesis
Oxidative phosphorylation
Chlorocyclohexane and chlorobenzene degradation
Photosynthesis - antenna proteins
Carbon fixation in photosynthetic organisms
01100 10/13/21
(c) Kanehisa Laboratories

MED4

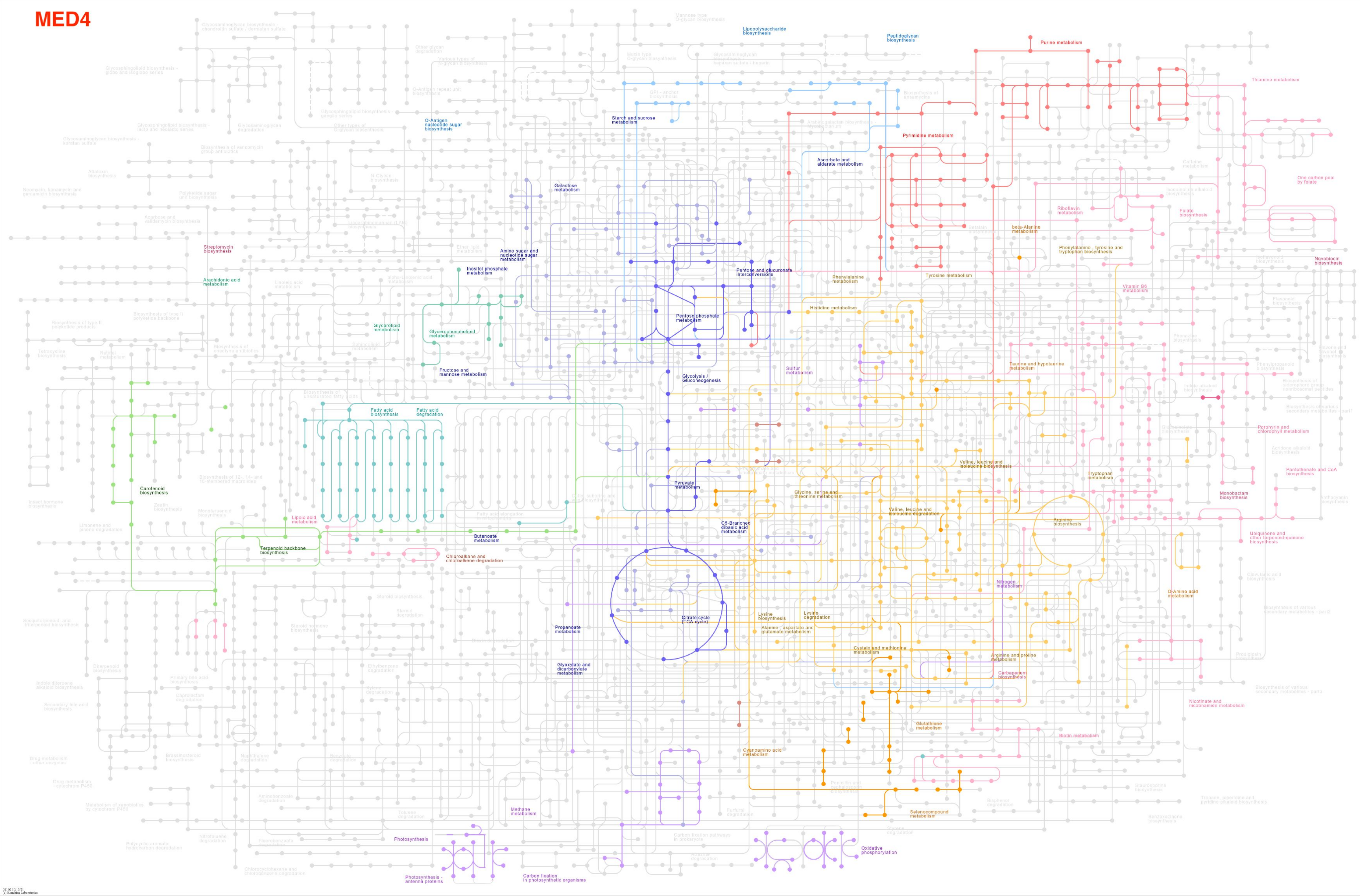

01100 10/13/21
(c) Kanehisa Laboratories

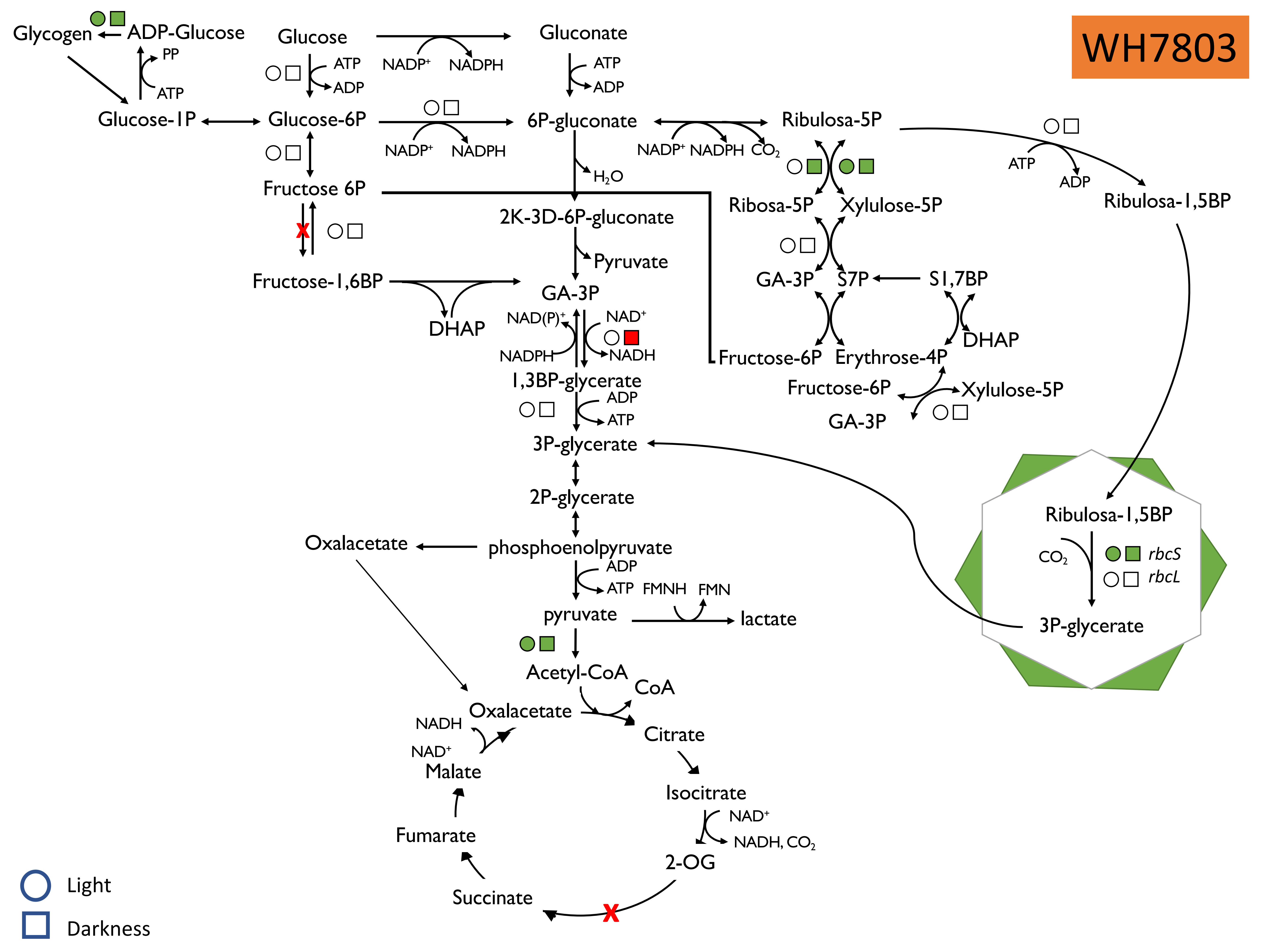
WH7803
Glycogen
ADP-Glucose
PP
ATP
Glucose-1P
Glucose
ATP
ADP
Glucose-6P
NADP+
NADPH
Gluconate
ATP
ADP
6P-gluconate
NADP+
NADPH
Fructose 6P
Fructose-1,6BP
DHAP
H2O
2K-3D-6P-gluconate
Pyruvate
GA-3P
NAD(P)+
NADPH
NAD+
NADH
1,3BP-glycerate
ADP
ATP
3P-glycerate
2P-glycerate
Oxalacetate
phosphoenolpyruvate
ADP
ATP
FMNH
FMN
pyruvate
lactate
Acetyl-CoA
CoA
Oxalacetate
Citrate
NADH
NAD+
Malate
Isocitrate
NAD+
NADH, CO2
Fumarate
2-OG
Succinate
Ribulosa-5P
NADP+ NADPH CO2
Ribosa-5P
Xylulose-5P
GA-3P
S7P
S1,7BP
DHAP
Fructose-6P
Erythrose-4P
Fructose-6P
Xylulose-5P
GA-3P
ATP
ADP
Ribulosa-1,5BP
Ribulosa-1,5BP
CO2
rbcS
rbcL
3P-glycerate
Light
Darkness

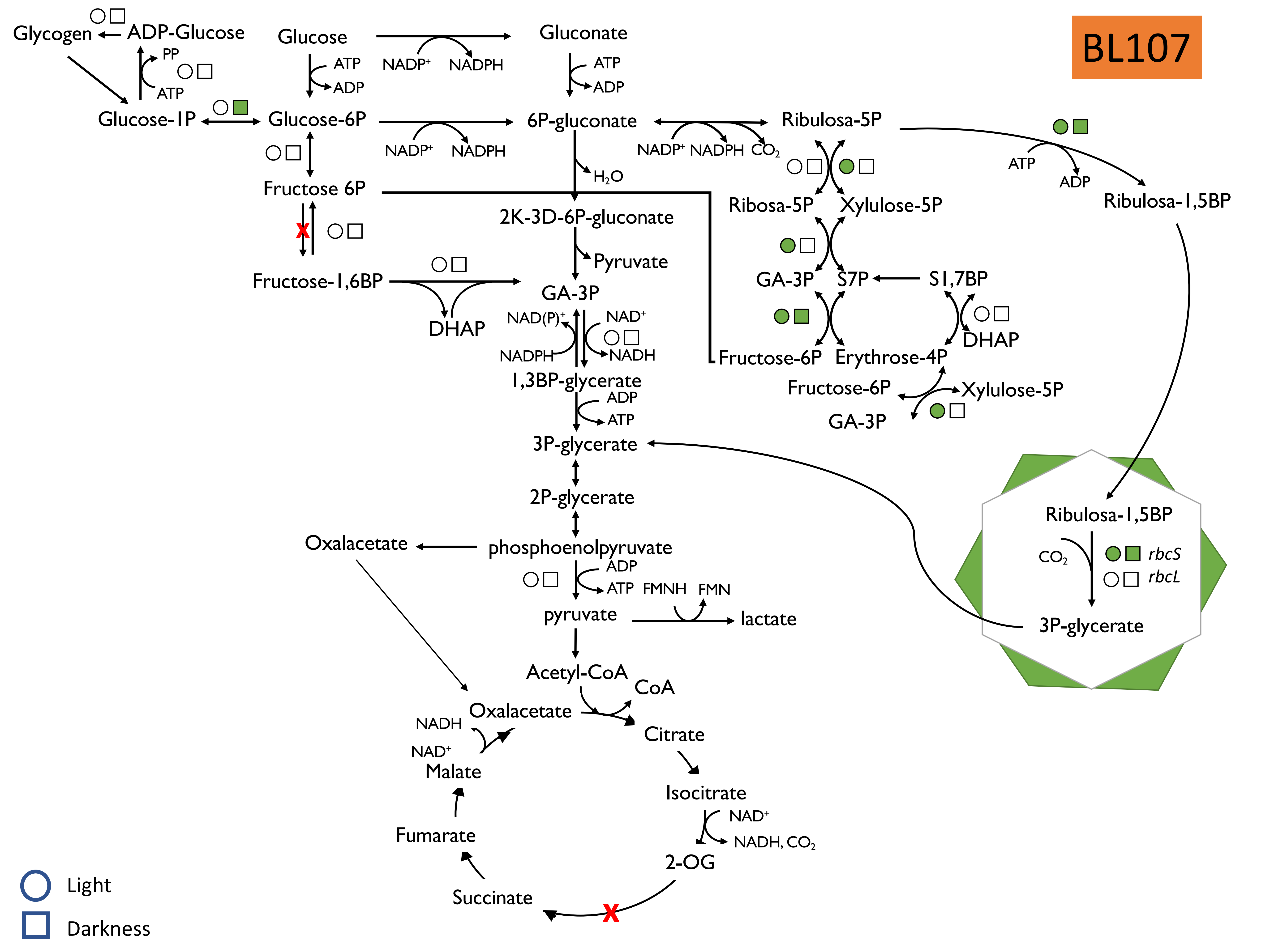
BL107
Glycogen
ADP-Glucose
PP
ATP
Glucose-1P
Glucose
ATP
ADP
Glucose-6P
NADP+
NADPH
Gluconate
ATP
ADP
6P-gluconate
NADP+
NADPH
CO2
Ribulosa-5P
ATP
ADP
Ribulosa-1,5BP
H2O
Fructose 6P
2K-3D-6P-gluconate
Pyruvate
Ribosa-5P
Xylulose-5P
GA-3P
S7P
S1,7BP
DHAP
Fructose-6P
Erythrose-4P
Fructose-6P
Xylulose-5P
GA-3P
Fructose-1,6BP
DHAP
GA-3P
NAD(P)+
NADPH
NAD+
NADH
1,3BP-glycerate
ADP
ATP
3P-glycerate
2P-glycerate
Oxalacetate
phosphoenolpyruvate
ADP
ATP
FMNH
FMN
pyruvate
lactate
Ribulosa-1,5BP
CO2
rbcS
rbcL
3P-glycerate
Acetyl-CoA
CoA
Oxalacetate
Citrate
NADH
NAD+
Malate
Isocitrate
NAD+
NADH, CO2
Fumarate
2-OG
Succinate
Light
Darkness

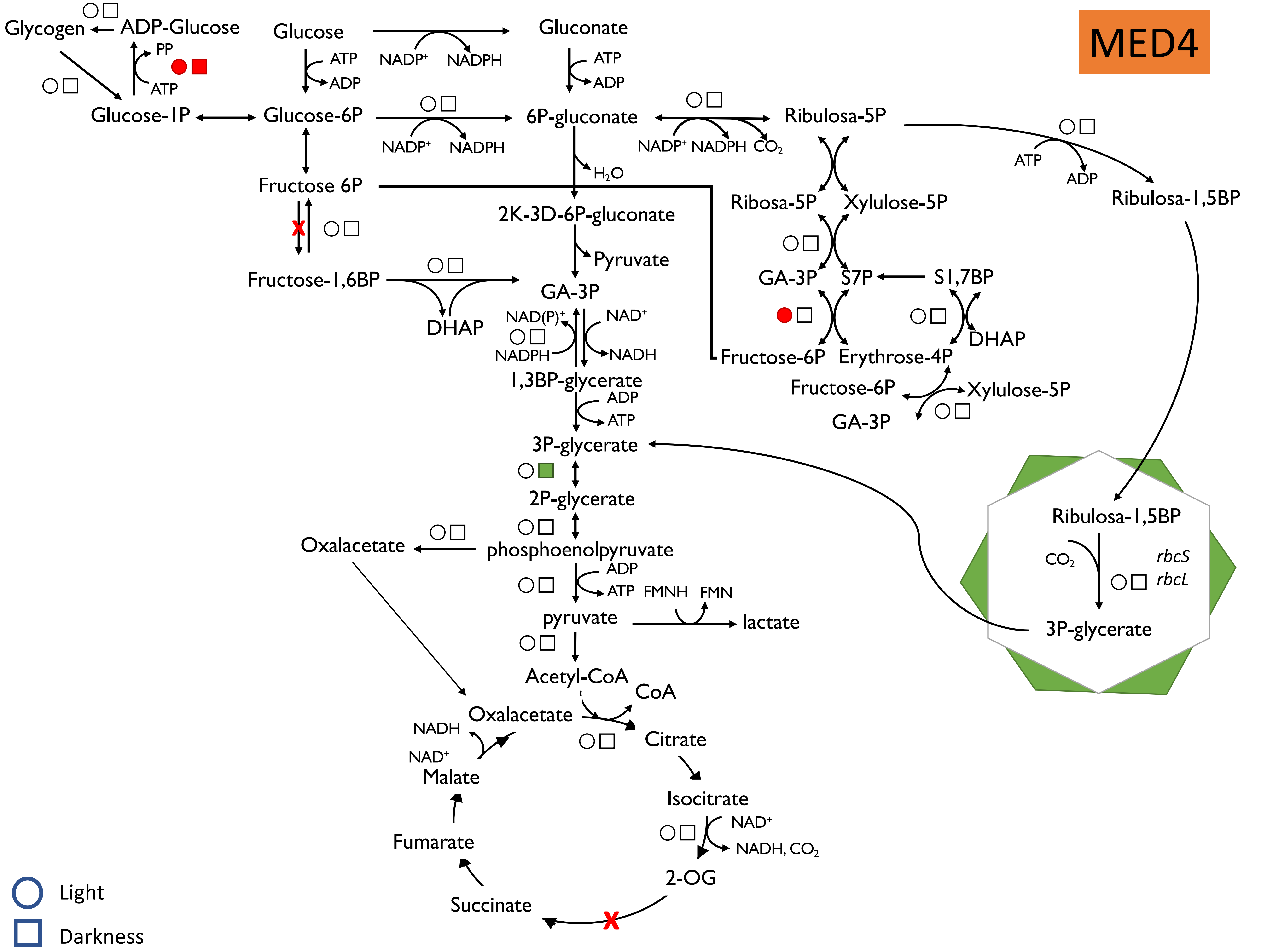
MED4
Glycogen
ADP-Glucose
PP
ATP
Glucose-1P
Glucose
ATP
ADP
Glucose-6P
NADP+
NADPH
Gluconate
6P-gluconate
H2O
Ribulosa-5P
CO2
Ribulosa-1,5BP
Fructose 6P
Fructose-1,6BP
DHAP
2K-3D-6P-gluconate
Pyruvate
GA-3P
NAD(P)+
NAD+
NADH
1,3BP-glycerate
3P-glycerate
2P-glycerate
phosphoenolpyruvate
Oxalacetate
pyruvate
FMNH
FMN
lactate
Acetyl-CoA
CoA
Citrate
Isocitrate
NADH, CO2
2-OG
Succinate
Fumarate
Malate
Ribosa-5P
Xylulose-5P
S7P
S1,7BP
Fructose-6P
Erythrose-4P
rbcS
rbcL
Light
Darkness

Legends for supplementary figures

Supplementary figure 1. Effect of glucose availability on the proportional abundance of genera in cultures from *Synechococcus* and *Prochlorococcus*. A. *Synechococcus* sp. WH7803 cultures. B. *Synechococcus* sp. BL107 cultures. C. *Prochlorococcus* sp. MED4 cultures. Proportional abundance was calculated based on total protein intensity per genera obtained by relative label-free abundance using MarRef v6 as database. Each column represents individual samples. CL=control light, D=Darkness, GmM= glucose 5 mM, GnM= glucose 100 nM. Three biological replicates were prepared per condition (1,2,3).

Supplementary figure 2. Global overview map highlighting quantified proteins in *Synechococcus* sp. strains WH8102 and WH7803, and *Prochlorococcus* sp. strain SS120 and MED4. The pathway map shows all the proteins quantified in the proteomic experiments performed with these cyanobacterial strains. These maps were created in R using the Pathview package (1). KEGG terms were retrieved using the specific tool in UniProt (www.uniprot.com). The *Synechococcus* sp. strain BL107 map is not shown since its genome has not been included yet in KEGG.

Supplementary figure 3. Summary of the proteomics results showing the statistically significant changes in enzymes involved in glucose metabolism in *Synechococcus* sp. strains WH8102 and BL107, and *Prochlorococcus* sp. strain under light and darkness. Circles, light; squares, darkness. Green colour indicates an increase in abundance, and red colour a decrease in abundance. Arrows indicate the metabolic flow upon glucose addition.

Legends for supplementary tables

Supplementary table 1. Compiled quantitation of changes in the proteomes of all studied cyanobacterial strains. Numbers highlighted in light green are statistically significant. TTEST.L.LGnM, Student's t-test of results from cultures in the light vs cultures in the light with 100 nM glucose; FC.L.LGnM, fold-change from control cultures in the light with 100 nM glucose/ cultures in the light ; TTEST.L.LGmM, Student's t-test of results from control cultures in the light vs cultures in the light with 5 mM glucose; FC.L.LGmM, fold-change from control cultures in the light with 5 mM glucose/ cultures in the light ; TTEST.D.DGnM, Student's t-test of results from cultures in darkness vs cultures in darkness with 100 nM glucose; FC.D.DGnM, fold-change results from cultures in darkness with 100 nM glucose/ cultures in darkness ; TTEST.D.DGmM, Student's t-test of results from control cultures in darkness vs cultures in darkness with 5 mM glucose; FC.D.DGmM, fold-change results from cultures in darkness with 5 mM glucose/ cultures in darkness ; TTEST.L.D, Student's t-test of results from cultures in the light vs cultures in darkness; FC.D/L, fold-change results from cultures in darkness / cultures in the light.

Supplementary table 2. Quantitation of changes in the proteomes of all studied cyanobacterial strains. Results for each cyanobacterial strain are shown in a separate tab. Abbreviations of column titles are the same as in Supplementary table 1.

Supplementary table 3. Quantitation of changes in the proteomes of *Alteromonas* from all studied cyanobacterial cultures. Results from each cyanobacterial culture are

shown in a separate tab. Abbreviations of column titles are the same as in Supplementary table 1.

Supplementary table 4. **Quantitation of changes in the proteomes of *Marinobacter* from all studied cyanobacterial cultures.** Results from each cyanobacterial culture are shown in a separate tab. Abbreviations of column titles are the same as in Supplementary table 1.

Bibliography

1. Luo W, Brouwer C. 2013. Pathview: an R/Bioconductor package for pathway-based data integration and visualization. Bioinformatics 29:1830-1.
